# Fabricating Inorganic/Organic S-Scheme Heterojunction for Efficient Photocatalytic Production of H_2_ and H_2_O_2_

**DOI:** 10.34133/research.1166

**Published:** 2026-03-03

**Authors:** Qinghong Cai, Haibo Zhou, Hongwen Zhang, Gaocan Li, Youzhou He, Fukun Li, Xingyan Liu, Siping Wei

**Affiliations:** ^1^ Engineering Research Center for Waste Oil Recovery Technology and Equipment, Ministry of Education, College of Environment and Resources, Chongqing Technology and Business University, Chongqing 400067, China.; ^2^ National Engineering Research Center for Biomaterials and College of Biomedical Engineering, Sichuan University, Chengdu 610064, China.; ^3^ Green Pharmaceutical Technology Key Laboratory of Luzhou City, School of Pharmacy, Southwest Medical University, Luzhou 646000, China.

## Abstract

Enhancing the efficiency of photocatalytic H_2_ evolution and H_2_O_2_ production through heterojunction engineering is crucial for addressing energy sustainability and environmental challenges. In this context, constructing S-scheme heterojunctions has emerged as a promising strategy. Here, we report an inorganic/organic S-scheme Bi_2_WO_6_/zinc(II) tetrakis(4-carboxy-phenyl)porphyrin (BWO/ZTP) heterojunction with a strong built-in electric field, constructed via an interface induction strategy. The optimal BWO/ZTP-1 achieves exceptional H_2_ evolution and H_2_O_2_ production activity, achieving 2,343.3 and 236.1 μmol·g^−1^·h^−1^. These represent remarkable enhancements of 14.9 and 3.44 times for H_2_ and 2.33 and 2.27 times for H_2_O_2_ over pristine BWO and Zn-TCPP. By using femtosecond transient absorption spectroscopy, Kelvin probe force microscopy, in situ x-ray photoelectron spectroscopy and density functional theory, we demonstrate that built-in electric field is pivotal for the exceptional performance, which leads to the proposal of a photocatalytic mechanism. This work provided feasible insights and references for the design of novel and superior inorganic/organic S-scheme heterojunction photocatalysts with tight contact and synergistic interaction for photocatalytic applications.

## Introduction

The escalating global energy demands driven by industrialization and population growth contrast sharply with the finite nature of conventional fossil fuel reserves [1,2]. The fossil fuel combustion generates detrimental environmental impacts, including excessive CO_2_ emissions, acid precipitation, and particulate matter pollution, collectively posing threats to ecological systems and public health. Thus, it has become urgent to find green, pollution-free, and renewable energy sources to replace fossil fuels . Hydrogen energy, as a high-efficiency, high-calorific value and environmentally friendly energy carrier, is considered an ideal alternative to fossil fuels [3]. As a chemical widely used in various industries, H_2_O_2_ is also a potential substitute for traditional fuels [4,5]. As highly efficient technology among a series of hydrogen production means, photocatalytic technology does not require external energy input or cause secondary pollution and has drawn widespread attention in fields such as energy conversion and environmental remediation [6–8]. Photocatalytic hydrogen evolution and H_2_O_2_ production, as a solar-powered and environmentally benign methodology, have emerged as a frontier research focus in sustainable fuel generation, requiring neither exogenous energy supply nor generating toxic by-products [9]. This approach could not only address the energy crisis but also reduce greenhouse gas emissions and ultimately foster sustainable development. The efficacy of photocatalytic water splitting critically depends on the development of advanced photocatalysts with optimized charge transfer dynamics [10]. The heterojunction is a very feasible strategy for the construction of highly efficient photocatalysts in recent years [11]. Among them, the S-scheme heterojunction has become a research hotspot not only as the rapid separation rate of photogenerated charge carriers but also as the reserved strong redox capacity.

The development of heterojunctions has evolved from the early type II to the Z-scheme and now to the current S-scheme [12]. In type II heterojunctions, the electrons and holes are concentrated in energy levels with relatively weak reduction and oxidation capabilities, which inherently diminishes the overall redox power. To address this issue, the concept of Z-scheme heterojunctions was proposed. However, their electron transfer pathway is considered thermodynamically challenging to achieve. Against this backdrop, the S-scheme heterojunction was introduced in 2019 [13]. A typical S-scheme heterojunction is composed of an oxidative photocatalyst (OP) and a reductive photocatalyst (RP), which are characterized by distinct energy band structures [14,15]. Upon contact, the difference in Fermi levels between the 2 semiconductors drives electron transfer from the material with the higher Fermi level to the one with the lower level until equilibrium is reached. This interfacial charge redistribution leads to the accumulation of opposite charges, which subsequently induces the formation of a built-in electric field (IEF) [16,17]. Under light irradiation, the IEF at the interface accelerates the recombination of photogenerated electrons from the OP with holes from the RP. This selective charge transfer mechanism effectively preserves holes with strong oxidative power in the valence band of the OP and electrons with high reductive potential in the conduction band of the RP, thereby enhancing the overall redox capability of the heterojunction system [18]. So far, the previously reported S-scheme heterojunctions are generally categorized into organic and inorganic types. All-inorganic S-scheme heterojunctions have good conductivity and carrier mobility and can produce electron–hole pairs after absorbing light energy, and these electron–hole pairs are easy to separate [19]. However, all-inorganic S-scheme heterojunctions often exhibit stringent fabrication requirements due to lattice matching constraints, which significantly limit their scalability [20,21]. In contrast, all-organic S-scheme heterojunctions show stronger adaptability and can be modified to have specific functions [22]. Unfortunately, all-organic S-scheme heterojunctions suffer from structural disorder, leading to charge carrier transport dominated by hopping between localized states that caused strong localization and shorter diffusion path [23,24]. According to the literature reported by Cheng et al. [25], in inorganic/organic system, the inorganic component typically possesses a stable and well-defined morphology, which could serve as a robust, high-quality substrate to induce the in situ growth of organic components at the interface, thus facilitating the rapid interfacial charge transfer. Meanwhile, the comfortably functionalized organic component with broad spectral response could effectively compensate for the inherently limited light-harvesting capability of the difficultly regulated inorganic counterpart [26]. More significantly, a key advantage of inorganic/organic heterojunctions lies in their superior stability and more efficient charge separation compared to all-inorganic or all-organic semiconductors, effectively addressing the limitations of the individual components while retaining strong redox capability [27]. The preparation methods of inorganic/organic heterojunction materials mainly include one-pot synthesis, in situ growth, self-template method, and so on. The in situ growth method is to grow one or more objects on the substrate under certain conditions. Compared with other methods, this method nucleates and forms on the substrate surface, so the matrix and guest molecules show excellent compatibility and tight interface strength [28]. On the basis of these considerations, we propose that the inorganic/organic S-scheme could enhance photocatalytic activity through synergistic interfacial interaction and optimized charge transfer pathways.

As a classical inorganic semiconductor, the bismuth tungstate (BWO) is characterized by a unique 2-dimensional (2D) layered architecture with excellent photochemical property [29]. Although the large specific surface area and abundant active sites provide a favorable platform for photocatalytic reactions, similar to many single-component semiconductors, BWO suffers from a relatively high recombination rate of photogenerated electron–hole pairs [30]. Coupling BWO with a 2D organic semiconductor to construct an S-scheme heterojunction could offer a promising strategy to promote charge carriers separation. Zinc(II) tetrakis(4-carboxy-phenyl)porphyrin (Zn-TCPP) nanosheets, a well-known metal–organic framework, are recognized as a 2D organic semiconductor with a large specific surface area and broad spectral response [31]. During the process of forming heterojunction, the 2D BWO provided a high-quality substrate for the nucleation and in situ growth of 2D Zn-TCPP, which facilitates the formation of an intimate interfacial contact and, in turn, accelerates interfacial charge carriers transfer. At the same time, considering the interleaved energy levels, 2D BWO and 2D Zn-TCPP are theoretically capable of forming a stable S-scheme heterojunction, which holds great promise for achieving superior photocatalytic performance.

An inorganic/organic S-scheme 2D/2D BWO/Zn-TCPP (BWO/ZTP) heterojunction was successfully fabricated through an in situ interface induction strategy in this study. Unlike most previously reported S-scheme heterojunction, the 2D Zn-TCPP was conformally in situ grown on the surface of 2D BWO, forming a more compact interface that facilitates rapid charge carriers transfer. Comprehensive analyses of crystallinity, morphology, photochemical, and electrochemical behaviors clearly confirmed the tight interfacial interaction between 2D Zn-TCPP and 2D BWO. The excellent photocatalytic activity of BWO/ZTP-1 was evaluated by photocatalytic hydrogen evolution (2,343.3 μmol·g^−1^·h^−1^) and H_2_O_2_ (236.1 μmol·g^−1^·h^−1^) experiments. Furthermore, the density functional theory (DFT) calculation was conducted to gain deeper mechanistic insight into the interfacial charge transfer process. The rational architecture engineering of inorganic/organic S-scheme 2D/2D heterojunction was conceptually advanced in this research, offering novel perspectives for promoting photocatalytic performance.

## Results and Discussion

### Characterization of catalysts

#### Crystal structure and composition analysis

**Fig. 1.**
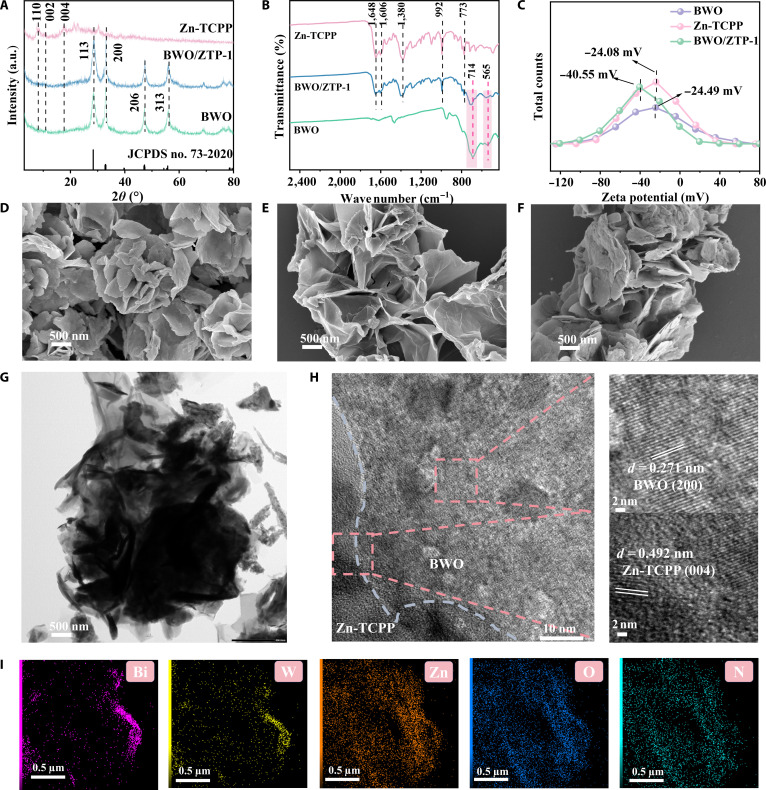
(A) XRD. a.u., arbitrary units. (B) FT-IR spectra. (C) Zeta potential profiles. (D to F) SEM of BWO, Zn-TCPP, and BWO/ZTP-1. (G and H) TEM and high-resolution TEM images. (I) Energy-dispersive x-ray mapping images of BWO/ZTP-1.

To determine the crystalline structure of the samples, we performed the x-ray diffraction (XRD) analysis on the samples over a scanning range from 5° to 80° at a rate of 6°·min^−1^. The resulting diffraction pattern was shown in Fig. 1A. The XRD pattern of BWO exhibited characteristic diffraction peaks at 28.68°, 33.02°, 47.52°, and 56.26°, which correspond to the (113), (200), (206), and (313) crystal planes [32,33]. These observations unambiguously confirmed the formation of highly crystalline BWO, as evidenced by its XRD pattern aligning with the standard diffraction data (JCPDS no. 73-2020). In addition, Zn-TCPP nanosheets exhibited distinct diffraction peaks at 2*θ* values of 7.96°, 10.75°, and 17.51°, which could be indexed to the (110), (002), and (004) crystal planes, respectively, thereby verifying the successful synthesis of Zn-TCPP nanosheets by aligning well with previously reported data [34,35]. It was worth noting that the characteristic peaks belonging to Zn-TCPP were not prominent in BWO/ZTP-1. The phenomenon may be attributed to the relatively low peak intensity of Zn-TCPP nanosheets compared to the stronger diffraction peaks of BWO, which could obscure the characteristic peaks of Zn-TCPP in the composite. In the locally enlarged Fig. S1A, weak peaks belonging to Zn-TCPP could be clearly observed in BWO/ZTP-1, indicating the preserved crystalline integrity of both BWO and Zn-TCPP. As could be seen in Fig. S1B, the intensity of the characteristic diffraction peaks of BWO in the composites gradually became stronger with the increase in BWO content. It could be preliminarily determined that there was a gradient change in the components of BWO in the composites.

To further verify the successful preparation of the BWO/ZTP, we conducted the Fourier transform infrared (FT-IR) analysis to characterize the functional groups of BWO, Zn-TCPP, and the resulting BWO/ZTP-1. As displayed in Fig. 1B, the original BWO exhibited 2 distinct characteristic peaks at 565 and 714 cm^−1^. The broad peak at 565 cm^−1^ attributed to Bi–O bonds and the peak at 714 cm^−1^ were associated with the stretching and bending vibrations of characteristic W–O bonds in BWO [36,37]. Notably, in the spectrum of BWO/ZTP-1, some minuscule shifts in the peaks at 565 and 714 cm^−1^ were observed. These shifts were likely due to strong interactions between BWO and Zn-TCPP nanosheets, which altered the electronic environment around the metal–oxygen bonds and resulted in changes in vibration frequency. Furthermore, as exhibited in Fig. S1C, the characteristic peaks of BWO became increasingly distinct with the rise in the content. In contrast to BWO, the characteristic peaks of Zn-TCPP were prominent in the BWO/ZTP. The peak at 773 cm^−1^ was primarily ascribed to the vibrations of the porphyrin ring skeleton, while the peak at 1,380 cm^−1^ corresponded to the stretching vibrations of the C═N bond. The vibration of the Zn–N bond resulted in a characteristic peak at 992 cm^−1^, indicating that Zn^2+^ successfully coordinated with the porphyrin [38]. The porphyrin–pyrrole ring’s stretching vibration led to the appearance of a peak at 1,606 cm^−1^, whereas the peak at 1,648 cm^−1^ arose from the stretching vibration of the carboxyl group’s C═O bonds [39]. Meanwhile, for BWO/ZTP-0.5 and BWO/ZTP-2, their XRD and FT-IR patterns differed from those of BWO/ZTP-1 only in peak intensity, while the peak positions remained the same, demonstrating that the crystal structure and functional groups of the materials remained unchanged. In addition, the zeta potential of the samples was measured to evaluate their stability (Fig. 1C), in which the BWO and Zn-TCPP exhibited zeta potential values of −24.49 and −24.08 mV. In contrast, the BWO/ZTP-1 presented a significantly more negative potential value of −40.55 mV. The pronounced alteration could likely be ascribed to the presence of Zn-TCPP, whose structure features 4 deprotonated carboxylate groups that collectively generate a surface rich in negative charges [40]. Zeta potential was a measure of the electrostatic repulsion or attraction between particles, for which the larger absolute zeta value indicated greater stability since the particles were less prone to aggregation upon dispersion. The high zeta potential value of BWO/ZTP-1 suggested that the formation of S-scheme heterojunction could significantly enhance stability.

#### Microscopic morphology and 2D structure analysis

The surface topography and nanostructure of BWO, Zn-TCPP, and BWO/ZTP-1 were examined via scanning electron microscopy (SEM). As demonstrated in Fig. 1D to F, the 2D BWO exhibited an uneven petal-like nanosphere appearance with a rough surface. Similarly, the 2D Zn-TCPP also displayed an analogous nanoflower-like morphology. However, the petals of Zn-TCPP were larger than those of BWO, offering a greater specific surface area, which facilitated the formation of encapsulated heterojunctions between the 2 components. Notably, the SEM images of BWO/ZTP-1 (Fig. 1F and Fig. S1D) revealed that the Zn-TCPP appeared to tightly cover on the surface of BWO, forming a distinctly tight self-assembling rather than a just respectively independent petal-like morphology. The result may be due to the fact that, as an inorganic component, the BWO had a stable and well-defined morphology, which provided a high-quality substrate for inducing the in situ growth of Zn-TCPP to form a dense BWO/ZTP heterojunction. The close contact between the BWO and Zn-TCPP was expected to enhance the transfer of photogenerated charge carriers. The transmission electron microscopy (TEM) and high-resolution TEM (Fig. 1G and H) further confirmed the above conclusion, in which the existence of the interface could be clearly observed. The lattice stripe spacings of 0.271 and 0.492 nm were observed on both sides of the interface, corresponding to the (200) crystal plane of BWO and the (004) crystal plane of Zn-TCPP, respectively [41]. Subsequently, the elements and composition of BWO/ZTP-1 were tested using energy-dispersive x-ray elemental mapping technique. It could be seen that N, O, Bi, W, and Zn were uniformly distributed on BWO/ZTP-1, confirming the heterojunction formation of BWO and Zn-TCPP.

**Fig. 2.**
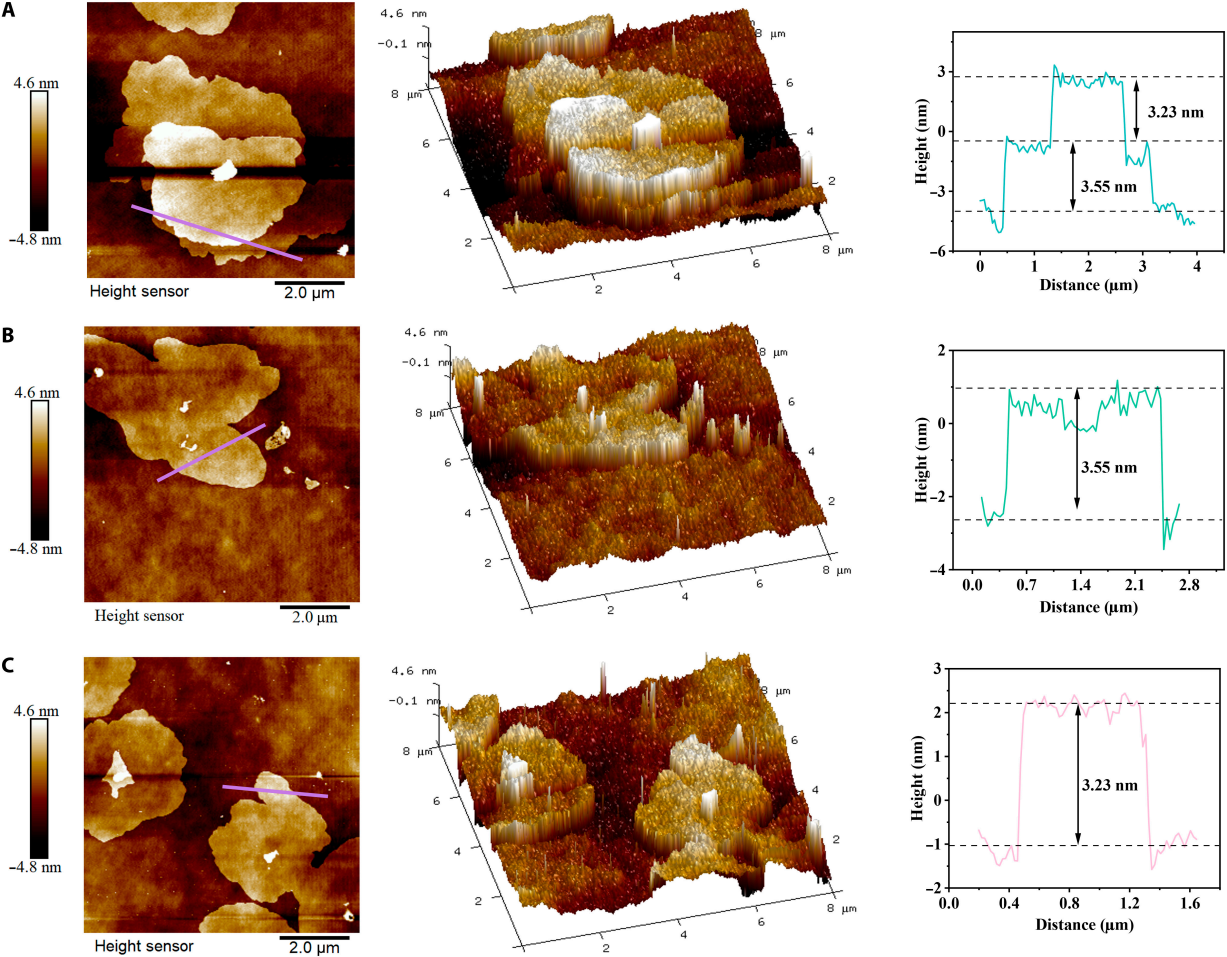
2D, 3D, and cross-sectional atomic force microscopy images of (A) BWO/ZTP-1, (B) BWO, and (C) Zn-TCPP.

The atomic force microscopy characterization was conducted to quantify the nanosheet thickness of samples. As revealed in Fig. 2, the nanosheet thicknesses of Zn-TCPP and BWO were measured as 3.23 and 3.55 nm, further corroborating the SEM results. In addition, the cross-sectional profile of BWO/ZTP-1 exhibited a step-like profile, where BWO served as the base layer and Zn-TCPP was attached to the surface in close contact. The results provided unambiguous evidence for the successful synthesis of the BWO/ZTP composite and established a foundation for constructing a dense heterojunction interface. Meanwhile, to verify the stacking form of Zn-TCPP on the surface of BWO, we physically mixed Zn-TCPP and BWO and then dispersed them in *N*,*N*′-dimethylformamide (DMF) along with BWO/ZTP to test their ultraviolet–visible (UV–vis) light absorption capability (Fig. S2A). Compared to physical mixing, the S-band of BWO/ZTP exhibited a blue shift, confirming the formation of face-to-face binding between the 2.

#### Specific surface area analysis

To comprehensively understand the impact of the structural characteristics for samples, we analyzed the N_2_ adsorption–desorption isotherms of BWO, Zn-TCPP, and BWO/ZTP-1 (Fig. S2B and C). As revealed in Fig. S2B, the adsorption–desorption isotherm of BWO exhibited a typical H_3_ hysteresis loop, corresponding to an type IV isotherm, which was indicative of a mesoporous structure. Conversely, the Zn-TCPP and BWO/ZTP-1 exhibited reversible type I adsorption–desorption profiles [42,43]. The specific surface areas and pore volume of the samples were summarized in Table S1. The Zn-TCPP showed a significantly larger specific surface area (125.94 m^2^·g^−1^) compared to BWO (17.35 m^2^·g^−1^), likely due to the larger wrinkled sheet structures of Zn-TCPP, as evidenced by the SEM image in Fig. 1D and E. For BWO/ZTP-1, the specific surface area (112.02 m^2^·g^−1^) was slightly reduced compared to original Zn-TCPP, which could be attributed to the in situ growth of Zn-TCPP on the BWO surface. This interaction potentially led to a more compact interface, resulting in a marginally decreased surface area for BWO/ZTP-1. In addition, the pore volume of BWO/ZTP-1 (0.084 cm^3^·g^−1^) was reduced compared to that of Zn-TCPP (0.138 cm^3^·g^−1^) and BWO (0.094 cm^3^·g^−1^), which confirms the close contact between the 2 monomers.

#### Analysis of element composition and chemical valence states

**Fig. 3.**
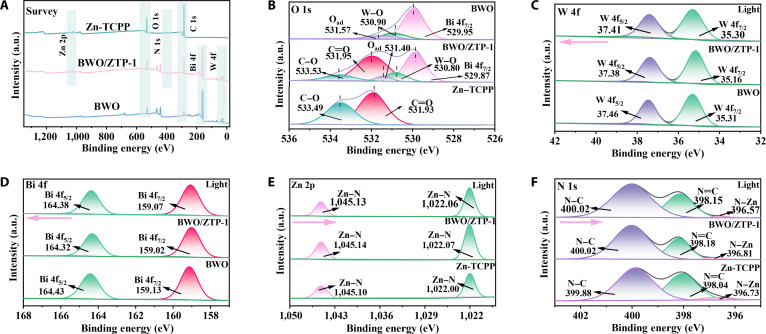
XPS of Zn-TCPP, BWO, and BWO/ZTP-1 (A) survey spectra. (B) O 1s XPS spectrum. XPS and in situ XPS of Zn-TCPP, BWO, and BWO/ZTP-1 (C) W 4f XPS spectrum. (D) Bi 4f XPS spectrum. (E) Zn 2p XPS spectrum. (F) N 1s XPS spectrum.

The chemical states of elements on the surfaces of BWO, Zn-TCPP, and BWO/ZTP-1 were characterized by x-ray photoelectron spectroscopy (XPS). The XPS survey spectrum (Fig. 3A) confirmed the presence of Zn, W, Bi, O, and N in the BWO/ZTP-1, indicating the successful combination of BWO and Zn-TCPP. The uniform distribution of elements across the surface ensured compositional consistency. The O 1s spectrum (Fig. 3B) showed peaks at 529.95, 530.90, and 531.57 eV, attributed to Bi–O, W–O and adsorbed oxygen (O_ad_) on the surface of BWO [44]. The peaks at 531.93 and 533.49 eV correspond to C═O and C–O bond components in Zn-TCPP. The presence of BWO and Zn-TCPP in BWO/ZTP-1 was confirmed. Compared to the O 1s binding energies of the 2 monomers, a shift occurred on the O 1s of the BWO/ZTP-1, resulting in a change in the binding energy. The peaks of Bi–O, W–O, and O_ad_ in the BWO/ZTP-1 were 529.87, 530.80, and 531.40 eV, respectively, and the peak positions were shifted toward lower binding energies compared to the monomer BWO, while the opposite was true for Zn-TCPP. This strongly confirmed the existence of interaction between the 2 monomers in the BWO/ZTP-1. These distinct oxygen species reflected the complex chemical environment of the BWO/ZTP-1 surface, which was expected to enhance the catalytic performance. In the W 4f spectrum (Fig. 3C), peaks at 35.31 and 37.46 eV corresponded to W 4f_7/2_ and W 4f_5/2_, which were characteristic of W^6+^ [45]. The peak positions of BWO/ZTP-1 appeared at 35.16 and 37.38 eV, again moving toward the low binding energy position. In the Bi 4f spectrum (Fig. 3D). The peaks of Bi 4f in BWO were located at 159.13 and 164.43 eV, corresponding to Bi 4f_7/2_ and Bi 4f_5/2_ [37]. The BWO/ZTP-1 was similarly shifted toward the lower field at 159.02 and 164.32 eV. The Zn 2p spectrum (Fig. 3E) showed peaks at 1,022.07 and 1,045.14 eV, corresponding to Zn–N bond (Zn 2p_3/2_ and Zn 2p_1/2_), which clearly confirmed the incorporation of Zn-TCPP in BWO/ZTP-1 [46]. The binding energy increased compared to the pristine Zn-TCPP (1,022.00 and 1,045.10 eV). It was possible that the chemical environment of Zn had changed because of the formation of composites, further suggesting a chemical interaction between Zn-TCPP and BWO. The N 1s spectrum (Fig. 3F) revealed peaks at 396.81, 398.18, and 400.02 eV, which corresponded to N–Zn, N═C, and N–C bonds, which indicated that BWO/ZTP-1 retained the structure feature in the Zn-TCPP. The binding energy shifted toward higher values suggested the formation of interfacial chemical bonds between Zn-TCPP and BWO, which altered the local electronic environment. The results further confirmed the interaction between BWO and Zn-TCPP, likely due to the formation of IEF, laying the foundation for the construction of a heterojunction. To further confirm the heterojunction scheme, we tested in situ XPS. The in situ XPS spectra of W, Bi, Zn, and N elements of BWO/ZTP-1 under illumination were shown in Fig. 3C to F. After illumination, the binding energy of W 4f and Bi 4f moved upward, and the binding energy of N 1s and Zn 2p moved to the lower position. The feature fully demonstrated the charge transfer from BWO to Zn-TCPP under illumination, which further confirmed that the S-scheme heterojunction formed between Zn-TCPP and BWO.

#### IEF analysis

**Fig. 4.**
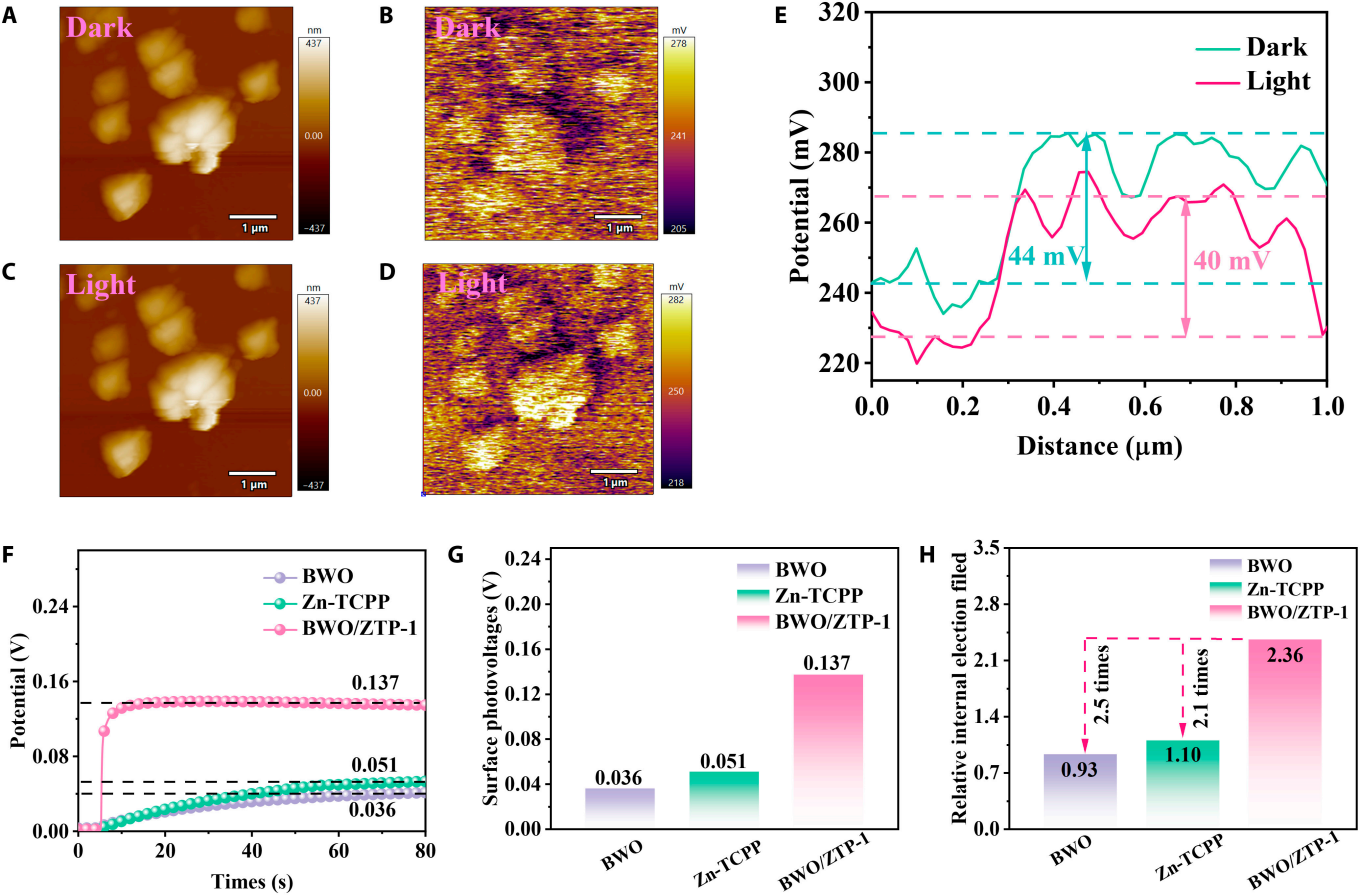
(A to D) KPFM in dark and light of BWO/ZTP-1. (E) Line scan surface potential of BWO/ZTP-1 under dark and light conditions. (F) Open-circuit voltage. (G) Surface potential and (H) IEF strength of BWO, Zn-TCPP, and BWO/ZTP-1.

As we all know, the IEF was crucial for accelerating the migration and separation of charge carriers. To verify the existence of the IEF and the spatial transfer of charges in BWO/ZTP-1, we used the Kelvin probe force microscopy (KPFM) to test the contact potential difference under dark and light conditions, as shown in Fig. 4A to E. Under dark conditions, a potential difference of 44 mV was detected, proving the existence of the IEF. After illumination, there was no significant change in the morphology of BWO/ZTP-1 (Fig. 4A to C), but the potential had changed. The overall decreased in surface potential indicated the accumulation of charges. Because of the coverage of Zn-TCPP on the surface of BWO, the decreased in potential under illumination indicated the aggregation of electrons in Zn-TCPP. This conclusion further confirmed the results of in situ XPS. To further verify the strength of the IEF, we performed the calculations based on the model proposed by Kanata-Kito et al. [47]. The surface potential and surface charge density were represented by the values of open-circuit voltage (Fig. 4F and G) and zeta (Fig. 1C), respectively. As shown in Fig. 4H of the calculation results, the IEF strength of BWO/ZTP-1 was 2.5 and 2.1 times higher than that of BWO and Zn-TCPP, respectively, which indicated that BWO/ZTP-1 could separate photogenerated carriers more efficiently. Meanwhile, the enhanced IEF intensity of this inorganic/organic S-scheme heterojunction also surpassed that of many reported materials to date (Table S2).

#### Optical property and band composition analysis

**Fig. 5.**
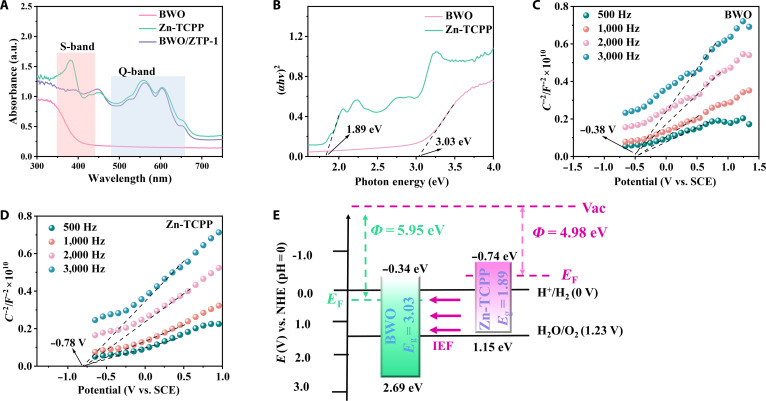
(A) UV–vis spectra of BWO, Zn-TCPP, and BWO/ZTP-1. (B) Tauc spectra of BWO and Zn-TCPP. (C and D) Mott–Schottky plots of BWO and Zn-TCPP. (E) Band structure diagram.

The optical absorption properties of BWO, Zn-TCPP, and BWO/ZTP-1 were characterized by UV–vis diffuse reflectance spectroscopy. As exhibited in Fig. 5A, BWO exhibited strong absorption in the range of 300 to 350 nm, with a gradual decline in absorption capacity beyond 350 nm. Compared to BWO, Zn-TCPP demonstrated significantly enhanced light absorption capacity, primarily attributed to the photosensitive porphyrin molecular unit, which contained multiple conjugated double bonds. These conjugated bonds enabled efficient photon absorption, leading to a strong S-band absorption peak near 380 nm and prominent Q-band absorption peaks in the range of 500 to 700 nm [48]. Upon the heterojunction formation, BWO/ZTP-1 also showed strong UV–vis light absorption, demonstrating the excellent light-harvesting performance for BWO/ZTP-1. The bandgaps (*E*_g_) of BWO and Zn-TCPP were calculated from the Tauc spectra (Fig. 5B), yielding the corresponding values of 3.03 and 1.89 eV, respectively. The energy band characteristic demonstrated that Zn-TCPP was more readily photoexcited to generate electron–hole pairs. Furthermore, the Mott–Schottky plots (Fig. 5C and D) at testing frequencies of 500, 1,000, 2,000, and 3,000 Hz exhibited positive slopes, indicating that both BWO and Zn-TCPP were n-type semiconductors. Further analysis of the Mott–Schottky plots revealed flat-band potentials of −0.38 and −0.78 V for BWO and Zn-TCPP, respectively. Typically, for n-type semiconductors, the conduction band potential (*E*_CB_) was about 0.1 to 0.3 V more negative than the flat-band potential. Therefore, the *E*_CB_ values of BWO and Zn-TCPP were estimated to be about −0.58 and −0.98 V. By converting these values into standard hydrogen electrode potentials using the formula *E*_NHE_ = *E*_SCE_ + 0.241 V, the potentials were determined to be −0.34 and −0.74 V, respectively. Finally, detailed information about the energy bands was derived on the basis of the Mott–Schottky plot, *E*_g_ values, and DFT data below, as shown in Fig. 5E. The energetic configuration analysis revealed that BWO and Zn-TCPP exhibited negative conduction band potentials (*E*_CB_ < 0 V), fulfilling the essential redox potential thresholds for proton reduction reaction. Notably, the more negative *E*_CB_ of Zn-TCPP indicated its superior photocatalytic hydrogen evolution capability. Moreover, the diagram also revealed staggered energy levels between BWO and Zn-TCPP, providing the basis for forming an S-scheme heterojunction.

**Fig. 6.**
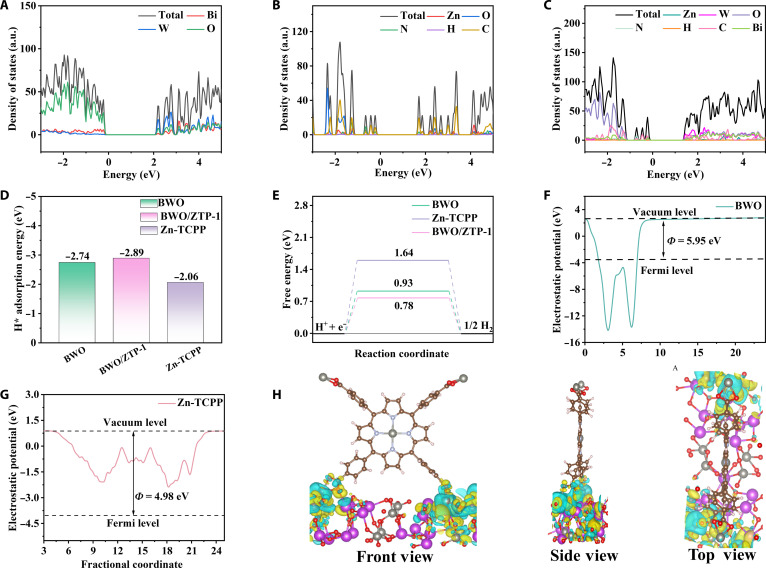
(A to C) DOS spectra of BWO, Zn-TCPP, and BWO/ZTP-1. (D) Energy for adsorption of H*. (E) Gibbs free energy of BWO, Zn-TCPP, and BWO/ZTP-1. Work function of (F) BWO and (G) Zn-TCPP. (H) Differential charge density plot of BWO/ZTP-1 under different views.

Theoretical calculations based on DFT were conducted for BWO, Zn-TCPP, and BWO/ZTP-1 to further investigate their energy band structures, as demonstrated in Fig. 6 and Fig. S3. The results revealed that the conduction band minimum and valence band maximum were located at the same position in *k*-space for all 3 samples, confirming that they were direct bandgap semiconductors . The calculated bandgap values were 2.3, 1.87, and 1.63 eV for BWO, Zn-TCPP, and BWO/ZTP-1 (Fig. S3A to C). Notably, the bandgap value of BWO/ZTP-1 decreased significantly compared to original BWO and Zn-TCPP, which indicated a wider photoresponse range. Furthermore, Fig. 6A and B presented the density of states (DOS) for BWO and Zn-TCPP, which were consistent with the energy band calculations. For BWO, the valence band was primarily contributed by O atom, reflected in the significant state density to the left of the Fermi level, while the conduction band was mainly derived from W and Bi atoms. While the valence band of Zn-TCPP was mainly contributed by the elements C, N, and O, the conduction band came from the elements C and Zn. Comparing the calculated bandgap with the experimental bandgap value, the difference between the 2 was not significant, further confirming the rationality of the calculated results. Figure 6C showed the DOS of the BWO/ZTP-1, in which the valence band was composed of O, C, and N atoms, while the conduction band consists of Bi, W, Zn, and C atoms. In addition, the energy for adsorption of H* and the Gibbs free energy Δ*G*_H*_ of the samples were also calculated, as shown in Fig. 6D and E. In general, the lower the value of adsorption energy, the easier the reaction. The values of adsorption energy for BWO, Zn-TCPP, and BWO/ZTP-1 were −2.74, −2.06, and −2.89 eV, respectively. The fact that BWO/ZTP-1 possessed the lowest value suggested that it had the highest adsorption for H atom, which was more favorable for the photocatalytic hydrogen evolution reaction. From the differential charge density plot of H* adsorption sites in Fig. S4A, when H atoms adsorb at the interface between BWO and Zn-TCPP, electron redistribution occurs. Meanwhile, the Δ*G*_H*_ values of BWO, Zn-TCPP, and BWO/ZTP-1 were 0.93, 1.64, and 0.78 eV, respectively. Usually, the interface effect and electronic structure optimization of heterojunction materials could significantly reduce the energy barrier of the reaction. The BWO/ZTP-1 had the lowest Δ*G*_H*_, which indicated that the formation of S-scheme heterojunction reduced the energy barrier and made the hydrogen evolution reaction easier.

**Fig. 7.**
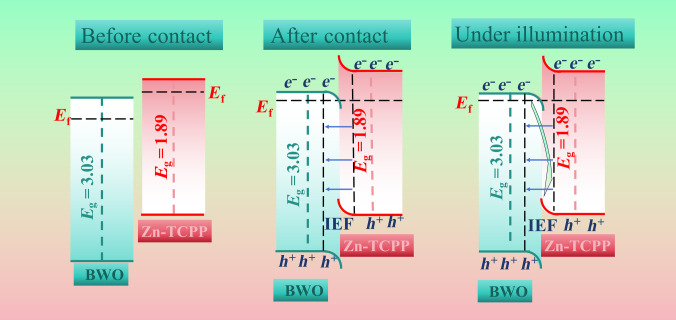
Interfacial electron transport pathways in S-scheme BWO/ZTP-1 heterostructure.

The work functions of BWO and Zn-TCPP, along with the charge density difference of the BWO/ZTP heterojunction, were calculated to ascertain the direction of charge flow. As displayed in Fig. 6F and G, the work functions of BWO and Zn-TCPP were 5.95 and 4.98 eV, respectively. The proposed charge transfer pathway (Fig. 7) was consistent with the fundamental principle of Fermi level alignment. It involved the spontaneous migration of electrons from the semiconductor with a higher Fermi level to its counterpart upon contact, a process initially driven by the concentration gradient. Specifically, as the BWO possessed a lower Fermi level compared to Zn-TCPP, electrons spontaneously transferred from Zn-TCPP to BWO until thermodynamic equilibrium was established, thereby achieving interfacial charge separation. The differential charge density in Fig. 6H and Fig. S4B revealed significant charge accumulation and dissipation localized at the BWO/ZTP heterointerface, with electron density redistribution predominantly occurring through interfacial migration from Zn-TCPP to BWO, in line with the work function calculation results. This directional charge transfer process will caused the energy band of Zn-TCPP to bend upward and the energy band of BWO to bend downward. More importantly, the retention of positive charges on the Zn-TCPP side and the accumulation of negative charges on the BWO side lead to the generation of IEF from Zn-TCPP toward BWO. Under light irradiation, photogenerated charge carriers migrated rapidly accelerated by the IEF, thereby promoting spatial charge carrier separation.

#### Photoelectrochemical analysis

**Fig. 8.**
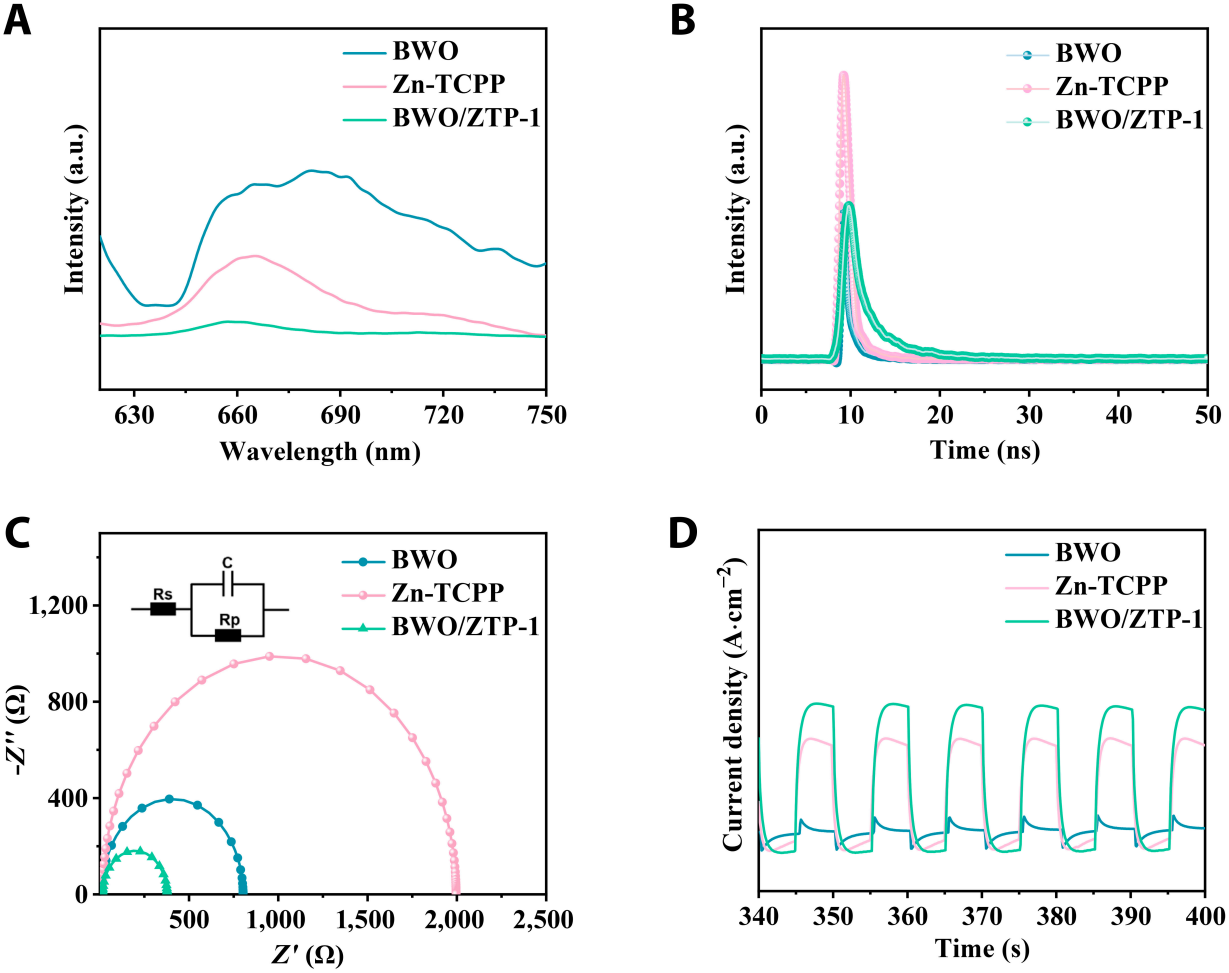
(A) The steady-state PL spectra. (B) Time-resolved PL. (C) Electrochemical impedance spectroscopy plots. (D) Photocurrent spectra of BWO, Zn-TCPP, and BWO/ZTP-1.

**Fig. 9.**
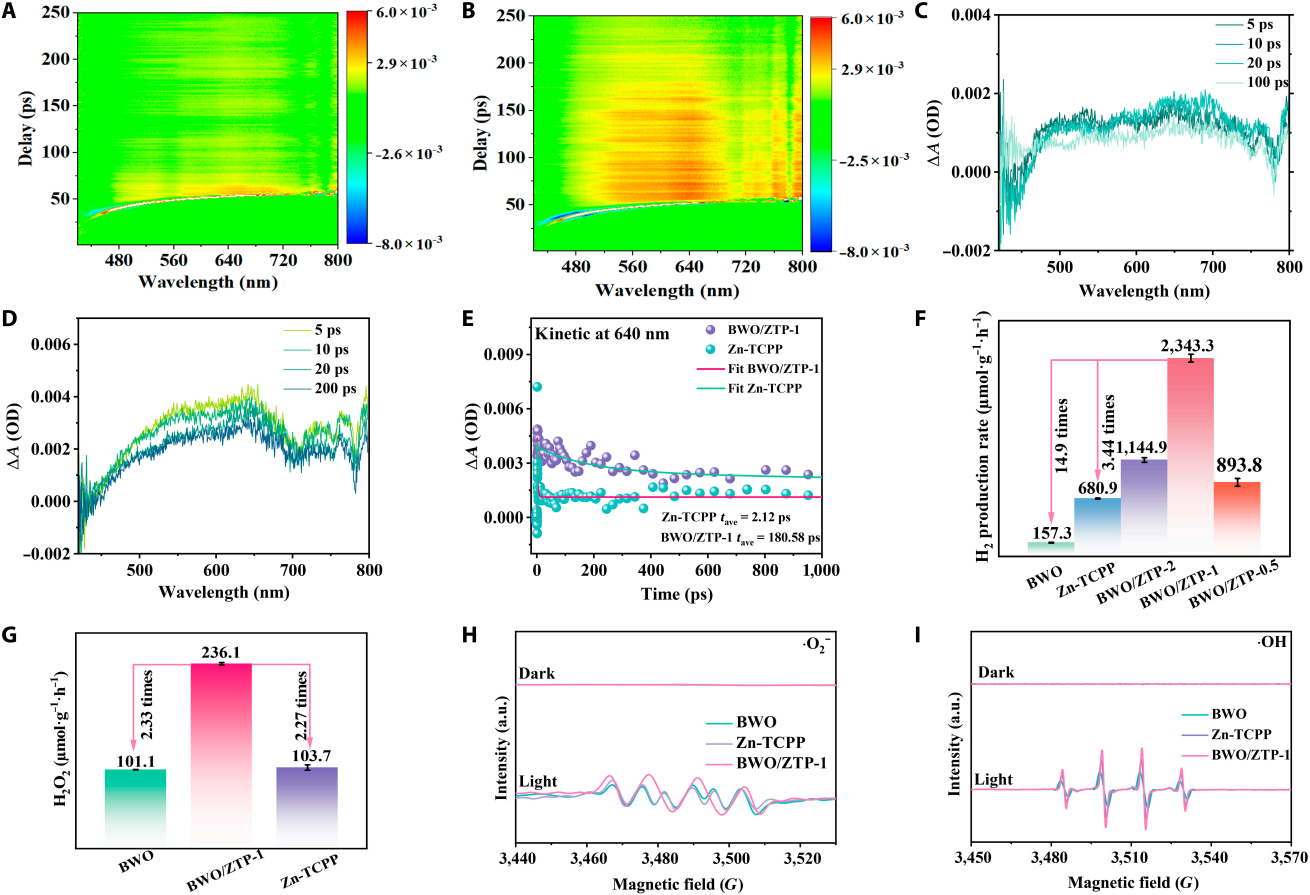
(A and B) 2D fs-TAS contour plots and (C and D) transient absorption spectra of Zn-TCPP and BWO/ZTP-1. (E) Normalized attenuation kinetics curves of transient absorption spectra of Zn-TCPP and BWO/ZTP-1 at 640 nm. (F) Photocatalytic hydrogen evolution rates of BWO, Zn-TCPP, BWO/ZTP-2, BWO/ZTP-1, and BWO/ZTP-0.5. (G) Photocatalytic H_2_O_2_ production of BWO, Zn-TCPP, and BWO/ZTP-1. (H and I) ESR spectrum of DMPO–•O_2_^−^ and DMPO–•OH.

The steady-state photoluminescence (PL) spectra systematically characterized charge carrier separation efficiency in BWO, Zn-TCPP, and BWO/ZTP-1, a key indicator for the principal determinant of photocatalytic activity. It was well known that the higher recombination rate of photogenerated electron–hole pairs corresponded to the higher PL peak intensity, indicating lower effective utilization of photogenerated charge carriers. As shown in Fig. 8A, the BWO exhibited a relatively high peak intensity, suggesting a high recombination rate of electron–hole pairs. While the Zn-TCPP demonstrated a slightly lower intensity compared to BWO, the separation efficiency of electron–hole pairs remained suboptimal. In contrast, the BWO/ZTP-1 displayed a significantly reduced peak intensity, attributed to the strong IEF of the S-scheme heterojunction, which accelerated the transfer of photogenerated charge carriers. The time-resolved PL spectroscopy (Fig. 8B) quantitatively assessed the photogenerated charge carrier separation capability. As tabulated in Table S3, the BWO and Zn-TCPP exhibited 0.40- and 0.49-ns average lifetime, while the BWO/ZTP-1 showed the extended durations of 1.05 ns. Apparently, the presence of S-scheme heterojunction accelerated the carrier transfer and thus improved the separation efficiency, resulting in the longest average lifetime of BWO/ZTP-1. The resistive property of semiconductors would directly influence the migration rate of photogenerated charge carriers in photocatalytic reactions, which was evaluated through electrochemical impedance spectroscopy. As depicted in Fig. 8C, the BWO/ZTP-1 exhibited a smaller semicircular arc radius compared to pristine BWO and Zn-TCPP, indicating a lower impedance and fast interfacial charge transfer rate. The photocurrent responses revealed charge separation and transfer more intuitively. As illustrated in Fig. 8D, the BWO demonstrated the weakest photocurrent response, while BWO/ZTP-1 displayed the strongest photocurrent response, which means that it had the best photogenerated carrier separation ability. Besides, the separation and migration dynamics of photogenerated charge carriers were investigated by femtosecond transient absorption spectroscopy (fs-TAS). From Fig. 9A to D, it could be visually observed that after photoexcitation, BWO/ZTP-1 exhibited a stronger excited-state absorption signal than Zn-TCPP, indicating superior charge separation ability. The attenuation kinetics curves of Zn-TCPP and BWO/ZTP-1 were shown in Fig. 9E. Compared with Zn-TCPP, the charge lifetime of BWO/ZTP-1 was significantly prolonged. The dynamic curve showed a trend of first rising and then falling, which indicated that the charge undergoes a process of formation and recombination. The charge lifetime of Zn-TCPP at 640 nm was 2.12 ps, indicating a very short survival time. However, BWO/ZTP-1 exhibited a charge lifetime of 180.58 ps at the same wavelength. This could be attributed to the presence of a huge IEF in the S-scheme heterojunction, which enabled efficient separation of photogenerated carriers [49].

### Photocatalytic activity tests

Photocatalytic hydrogen evolution experiments were conducted under a 300-W Xe lamp with a 420-nm cutoff filter and a light power density of 525 mW·cm^−2^ to evaluate the photocatalytic performance of the samples. As exhibited in Fig. 9F, the hydrogen production rates for BWO and Zn-TCPP were 157.3 and 680.9 μmol·g^−1^·h^−1^, while BWO/ZTP-1 achieved a significantly higher rate of 2,343.3 μmol·g^−1^·h^−1^, which was 14.9 and 3.44 times greater than those of original BWO and Zn-TCPP, and the performance was superior to previously reported composite materials based on BWO (Table S4). The remarkable improvement was attributed to the efficient IEF of the 2D/2D S-scheme heterojunction, which accelerated the transfer of photogenerated electron–hole pairs and enabled efficient charge carriers separation. In addition, the effect of BWO/ZTP via varying the theoretical mass ratios on hydrogen production was investigated. Experimental results indicated that an optimal H_2_ evolution rate was 1,144.9 μmol g^−1^·h^−1^ at BWO:Zn-TCPP = 2:1 mass ratio, while the H_2_ evolution rate declined to 893.8 μmol g^−1^·h^−1^ when Zn-TCPP content dominated (BWO:Zn-TCPP = 1:2). The reduction in efficiency with an increase in Zn-TCPP content was likely due to the relatively weaker interaction between the 2 components, resulting in insufficient formation of tightly connected S-scheme heterojunction. Conversely, when BWO was in excess, agglomeration occurred, reducing the number of active sites and hindering hydrogen production efficiency. These findings emphasized the critical role of the mass ratio in tightly forming an efficient IEF to facilitate charge transfer. To evaluate the stability of the material, we conducted the long-term stability testing, and from the first cycle to the fourth cycle, the hydrogen production efficiency of BWO/ZTP-1 did not significantly decreased (Fig. S5). XRD, XPS, SEM, and FT-IR characterization of the BWO/ZTP-1 after the fourth cycle (Fig. S6) revealed no notable changed in structure, demonstrating excellent stability and potential for practical applications. In addition, Fig. S7 illustrated the relationship between the apparent quantum efficiency (AQE) and solar-hydrogen conversion efficiency (STH) of BWO/ZTP-1 at specific wavelengths (400, 420, and 500 nm). The variation of AQE and STH was the same as that of UV–vis spectroscopy. Among them, the AQE and STH were exhibited at 420 nm, which were 1.03% and 0.43%, respectively. Table S5 listed a series of reported AQE data for BWO-based and Zn-TCPP-based photocatalysts. It could be observed that BWO/ZTP-1 had superior AQE, which may be due to the integration of BWO’s excellent conductivity and Zn-TCPP’s excellent photosensitivity, enabling it to better utilize sunlight.

To further evaluate the photocatalytic performance of the materials, we carried out the experiment of photocatalytic production of H_2_O_2_. As shown in Fig. 9G, the H_2_O_2_ production rates of BWO and Zn-TCPP were 101.1 and 103.7 μmol·g^−1^·h^−1^, respectively. The rate of BWO/ZTP-1 was 236.1 μmol·g^−1^·h^−1^, which was 2.33 and 2.27 times higher than that of BWO and Zn-TCPP. Meanwhile, after 4 cycles, the rate of H_2_O_2_ generation did not significantly decrease, and the XRD and XPS (Fig. S6) of the recovered material showed no significant changes, which confirmed its excellent stability. To confirm the path of photocatalytic H_2_O_2_ production, we carried out tests under different conditions (Fig. S8). The H_2_O_2_ yield of the system decreased significantly when Ar was introduced, which confirmed the importance of O_2_ in the reaction. At the same time, when AgNO_3_ was added as an electron sacrificial agent, almost no H_2_O_2_ was produced, which confirmed the importance of electrons in the reaction again. When *para*-benzoquinone was added as a sacrificial agent for ·O_2_^−^, the yield of H_2_O_2_ in the system significantly decreases, confirming the importance of ·O_2_^−^ in the reaction. Then, according to the electron spin resonance (ESR) results, BWO/ZTP-1 had enhanced ability to produce ·O_2_^−^, which could be confirmed that the generation of H_2_O_2_ was through 2-step one electron oxygen reduction (O_2_→·O_2_^−^→H_2_O_2_).

### Possible photocatalytic mechanism

**Fig. 10.**
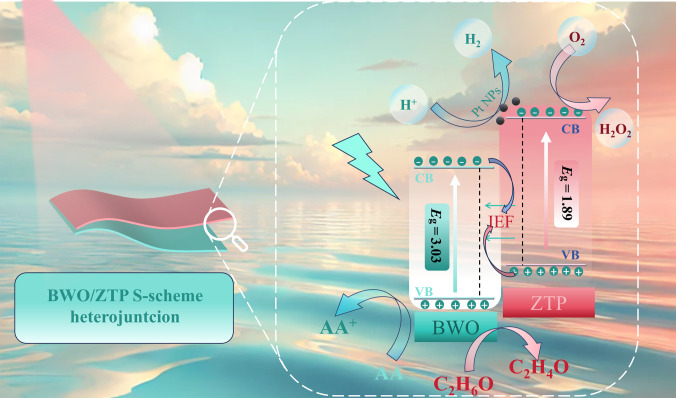
Schematic of photocatalytic mechanism in BWO/ZTP. VB, valence band; NPs, nanoparticles.

To further investigate the possible catalytic mechanism of the BWO/ZTP-1 heterojunction, we performed the radical species detection via ESR signal analysis to characterize the redox capacity of samples. As shown in Fig. 9H and I, no characteristic signals of 5,5-dimethyl-1-pyrroline *N*-oxide (DMPO)–·O_2_^−^ were detected for BWO, Zn-TCPP, and BWO/ZTP-1 without light irradiation; however, postillumination (15 min), the BWO/ZTP-1 composite exhibited the strongest DMPO–·O_2_^−^ signals, indicating its superior reduction capability for proton reduction to generate hydrogen. Furthermore, the ESR signals of DMPO–·OH followed a similar trend. Almost no ·OH radicals were observed in the dark, whereas the BWO/ZTP-1 heterojunction displayed the most intense DMPO–·OH signals under illumination, further confirming its exceptional oxidation capacity. In addition, the oxidation ability of BWO/ZTP-1 was further validated through sacrificial agent experiments, as shown in Fig. S9. By adjusting the dosage of sacrificial agent (l-ascorbic acid), a significant change in hydrogen production rate could be observed. When no l-ascorbic acid was added, almost no H_2_ was produced, and the yield of H_2_ increased significantly with the increase in l-ascorbic acid. When 1 M l-ascorbic acid was added, the rate of H_2_ generation reached 1,978.3 μmol·g^−1^·h^−1^. When the dosage of l-ascorbic acid was 2 M, the rate of 2,343.3 μmol·g^−1^·h^−1^ did not change significantly compared to the addition of 1 M sacrificial agent, which indicated that the hydrogen production rate reaches its maximum value. These ESR results provided very compelling evidence that the S-scheme heterojunction could effectively preserve the strong redox property. On the basis of the above discussion, we proposed a feasible photocatalytic mechanism for BWO/ZTP-1, as illustrated in Fig. 10. When BWO and Zn-TCPP come into contact, the difference in Fermi level could cause a redistribution of charges. The migration of charges caused band bending and the formation of IEF. Under light irradiation, both BWO and Zn-TCPP could absorb photons and generate photogenerated electron–hole pairs. The IEF provided strong driving force, causing charges and holes to migrate in opposite directions, effectively achieving spatial separation of electron–hole pairs. The photogenerated holes retained in BWO exhibited very strong oxidative capability, which could effectively oxidize l-ascorbic acid. Meanwhile, the photogenerated electrons remaining in the Zn-TCPP possessed high reductive potential, enabling the reduction of H^+^ to H_2_. In this system, the H_2_PtCl_6_ in situ photoreduced to Pt nanoparticles was introduced as a cocatalyst, which acted as efficient electron traps, forming active catalytic sites that further facilitate the reduction of H^+^ to H_2_. The l-ascorbic acid was used as a sacrificial agent to scavenge photogenerated holes, producing dehydroascorbic acid and suppressing electron–hole recombination. This process provided a stable reductive environment for H^+^ to H_2_, ensuring efficient hydrogen evolution.

## Conclusion

In summary, an inorganic/organic S-scheme BWO/ZTP heterojunction was fabricated via an in situ interface induction strategy. Among these samples, the BWO/ZTP-1 exhibited a remarkable hydrogen evolution efficiency and H_2_O_2_ production activity, achieving 2,343.3 and 236.1 μmol·g^−1^·h^−1^. The H_2_ production capacity of BWO/ZTP-1 was 14.9 and 3.44 times, and the H_2_O_2_ production capacity was 2.33 and 2.27 times that of BWO and Zn-TCPP. Furthermore, the photocatalytic activity of BWO/ZTP-1 remained stable with no significant decline after consecutive 4 cycles. The outstanding catalytic performance observed in BWO/ZTP-1 likely originated from the well-constructed S-scheme heterojunction system. Through fs-TAS, KPFM, and in situ XPS characterization techniques combined with DFT calculations, it was confirmed that the S-scheme heterojunction of BWO/ZTP-1 exhibited enhanced carrier mobility and robust redox capability, enabling efficient photocatalytic hydrogen evolution and H_2_O_2_ production. This work offered very valuable insights and references for the design and fabrication of inorganic/organic S-scheme heterojunction for photocatalytic application.

## Materials and Methods

### Materials

All chemical reagents were used as received without additional purification. Zinc nitrate hexahydrate [Zn(NO_3_)_2_·6H_2_O, 98%], bismuth nitrate pentahydrate [Bi(NO_3_)_3_·5H_2_O, 99%], pyrazine (C_4_H_4_N_2_, 99%), sodium tungstate dihydrate (Na_2_WO_4_·2H_2_O, 99.5%), and polyvinylpyrrolidone were procured from Alfa Aesar. Adamas supplied pyrrole (C_4_H_5_N, 99%), methyl 4-formylbenzoate (C_9_H_8_O_3_, 98%), and chloroplatinic acid (H_2_PtCl_6_, 98%). Solvents and auxiliaries including methanol (CH_3_OH, 99.9%), tetrahydrofuran (C_4_H_8_O,98%), propionic acid (C_3_H_6_O_2_, 99.5%), ethylene glycol (C_2_H_6_O_2_, 98%), *N*,*N*-dimethylformamide (C_3_H_7_NO, 98%), potassium hydroxide (90%), and ethanol (C_2_H_6_O, 98%) were originated from Chron Chemicals.

### Synthesis of samples

#### Synthesis of Zn-TCPP

First, 4.5 mg of Zn(NO_3_)_2_·6H_2_O, 0.8 mg of C_4_H_4_N_2_, and 20 mg of polyvinylpyrrolidone were dissolved in a mixed solution of 9 ml of DMF (*V*_1_) and 3 ml of ethanol (*V*_2_) (*V*_1_:*V*_2_ = 3:1), designated as solution A. Separately, 4 mg of TCPP was dissolved in 3 ml of DMF and 1 ml of ethanol with the same volume ratio of 3:1, designated as solution B. Under continuous stirring, the solution B was added dropwise to the solution A. Following the addition, the mixture was stirred (5 min) and subjected to ultrasonic treatment (10 min). A precise aliquot (16 ml) of the resultant solution was then sealed in glass vials and reacted at 80 °C for 16 h. After cooling naturally, the product was washed with ethanol (×3) and vacuum-dried at 60 °C for 10 h.

#### Synthesis of BWO

Initially, 0.97 g of Bi(NO_3_)_3_·5H_2_O and 0.6 g of cetyltrimethylammonium bromide were dissolved in 60 ml of deionized water with stirring for 30 min to ensure thorough mixing. Subsequently, 0.33 g of Na_2_WO_4_·2H_2_O was introduced, and the solution was stirred continuously for additional 2 h. The mixture was subjected to a hydrothermal treatment at 180 °C for 12 h in a 100-ml Teflon-lined autoclave. After natural cooling to room temperature, the resulting product was isolated by centrifugation, sequentially washed with anhydrous ethanol and deionized water (3 times each), and finally vacuum-dried at 60 °C for 10 h to yield the final product, designated as BWO.

#### Synthesis of BWO/ZTP

**Fig. 11.**
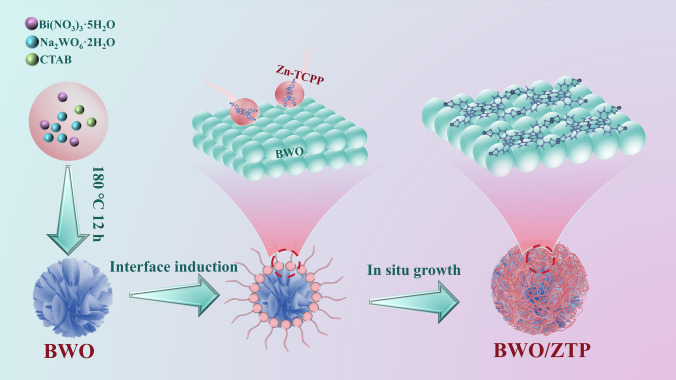
Synthetic pathway for 2D/2D BWO/ZTP assembly.

The preparation of the composite materials was similar to the synthesis method of Zn-TCPP nanosheets Fig. 11. BWO was uniformly dispersed in 1 ml of ethanol at different theoretical mass ratios and sonicated for 10 min to obtain solution C. After thoroughly mixing solutions A and B, solution C was added, and the remaining steps were identical to those used for Zn-TCPP synthesis. On the basis of the theoretical mass ratios of BWO and Zn-TCPP (BWO:Zn-TCPP = 0.5, 1, and 2), the final products could be named BWO/ZTP-0.5, BWO/ZTP-1, and BWO/ZTP-2.

## Data Availability

Data will be made available on request.
