# Supplementary material for: Fabricating Inorganic/Organic S-Scheme Heterojunction for Efficient Photocatalytic Production of H2 and H2O2
Source: Research (Wash D C). 2026 Mar 3;9:1166. doi: 10.34133/research.1166 (PMC12954277; doi:10.34133/research.1166)
Supplement: Supplementary 1 — Supplementary Text Figs. S1 to S9 Tables S1 to S5 [file research.1166.f1.zip › Supplementary Information.docx]

**Fabricating inorganic/organic S-scheme heterojunction for efficient photocatalytic production of H_2_ and H_2_O_2_**

**1. Experimental section**

**1.1 Synthesis of tetrakis(4-carboxyphenyl)porphyrin complex (TCPP)**

The tetrakis (4-carboxyphenyl) porphyrin (TCPP) was synthesized with reference to reported methods, with slight modifications.

(1) 5, 10, 15, 20-tetrakis (4-methoxycarbonylphenyl) porphyrin (TMCPP).

Methyl 4-formylbenzoate (14.410 g, 0.086 mol) was completely dissolved in propionic acid (250 mL) in a 500 mL round-bottom flask, pyrrole (6.09 mL, 0.086 mol) dissolved in 20 mL propionic acid was added slowly, then the mixture was refluxed at 150 °C for 12 hours. After cooling to room temperature, the obtained precipitate was washed with a large amount of ethanol, ethyl acetate and a small amount of THF, successively. The collected purple precipitate was dried at 60 °C for 12 hours.

(2) 5, 10, 15, 20-tetrakis(4-carboxyphenyl)porphyrin (H_2_TCPP).

Put TMCPP (500 mg) into the 250 mL round bottom flask with 40 mL THF and 40 mL MeOH, and 40 mL of water dissolved with 3 g KOH was added, then reflux the mixture solution at 90 ℃ for 12 h. After cooling to room temperature, part of the solvent was removed, then filtered to obtain a clear solution, subsequently the solution was acidified with 1 M HCl until no more precipitate was formed (pH ≈ 2), finally the collected precipitate was obtained via centrifugation and washed with water several times and dried at 80 ℃ under vacuum for 8 hours

**2. Characterization**

The crystallographic structure of the materials was characterized using X-ray powder diffraction (XRD) patterns (XRD-6100, Shimadzu, Japan). The microstructure of the samples was examined by scanning electron microscopy (SEM) (Zeiss Merlin, Germany) and transmission electron microscopy (TEM) (Talos F200S, USA). Additionally, the layer thickness and changes in surface potential of the materials was further characterized by atomic force microscopy (AFM) (Dimension Icon, Bruker, USA; Oxford Instruments, Jupiter XR, UK). The functional groups present in the samples were identified by Fourier-transform infrared spectroscopy (FT-IR) (Nicolet Apex, Thermo Scientific, USA). The pore structure and specific surface area of the samples were analyzed using Brunauer-Emmett-Teller (BET) method (ASAP 2020, USA). The stability of the samples in aqueous systems was assessed by Zeta potential analysis (Zetasizer Advance, Malvern Panalytical, UK). The dynamic migration of charge carriers was analyzed using femtosecond transient (fs-TAS) absorption spectroscopy (Ultrafast sytem, Helios, USA). The composition and chemical states of elements in the samples were characterized by X-ray photoelectron spectroscopy (XPS) and *in-situ* X-ray photoelectron spectroscopy (*in-situ* XPS) (Thermo Scientific K-Alpha, USA; Thermo ESCALAB nexsaG2, USA). The reducing capability of the samples was tested by electron spin resonance (ESR) (JES-FA200, Hitachi, Japan) to elucidate the reaction mechanism. The electron separation and migration abilities of the samples were characterized using photoluminescence spectrophotometer (PL) (F-7000, Hitachi, Edinburgh Instruments) and time-resolved photoluminescence spectroscopy (TRPL) (Edinburgh FS5, UK). The photoresponse of the samples was studied using UV-visible diffuse reflectance spectroscopy (UV-Vis DRS, Shimadzu, Japan). The electrochemical properties of the samples were determined by testing transient photocurrent response curves, Mott-Schottky plots and Tafel curves (PEC 1000, Perfect Light, China) and electrochemical impedance spectroscopy (EIS) (Electrochemical Workstation, CHI660E).

**3. Electrochemical measurements**

The electrochemical performance of the material was investigated using an electrochemical workstation (CHI660E) equipped with a three-electrode system. The three-electrode setup included a saturated calomel electrode as the reference electrode, a platinum foil as the counter electrode and an ITO glass coated with the sample as the working electrode. First, the 2 mg sample was weighed and dispersed in 1 ml ethanol under ultrasonic conditions to achieve uniform dispersion, followed by the addition of 10 µL Nafion solution. Using a pipette, 100 µL of this dispersion was accurately taken and evenly spread onto the surface of the ITO glass to prepare the working electrode. During testing, a 300 W Xe lamp was used as the light source and a 0.2 M Na_2_SO_4_ solution served as the electrolyte. Photocurrent response, Mott-Schottky plots, Tafel curves and electrochemical impedance spectroscopy (EIS) were used for electrochemical characterization.

**4. Photocatalytic H_2_ generation experiments**

The photocatalytic hydrogen production reaction was conducted under a 300 W Xe lamp equipped with a 420 nm cutoff filter, with a light power density of 525 mW/cm^2^, for a duration of 4 hours. Specifically, 10 mg photocatalyst was weighed and uniformly dispersed in 45 mL ultrapure water under stirring conditions, followed by the addition of 880 mg ascorbic acid (2 M) as a sacrificial agent and 2 wt% Pt as co-catalysts. Subsequently, nitrogen was bubbled through the well-dispersed suspension for 15 minutes to completely remove dissolved oxygen. A temperature-controlled cooling circulation pump was used to maintain the reaction temperature at approximately 5 °C. Finally, nitrogen was used as the carrier gas and hydrogen production was quantified by collecting data every 30 minutes using gas chromatography (GC-7920).

To measure the apparent quantum efficiency (AQE), all other conditions were constant, with only the 420 nm cutoff filter replaced by a 420 nm monochromatic filter, and the experiment was conducted for 4 hours. The AQE was calculated using the following formula:

$$AQE=\frac{2\times Numberofevolvedhydrogenmolecules}{Numberofincidentphotons}\times100\%=\frac{2N_{A}R}{E\lambda/hc}$$

In the formula, R represents hydrogen production (mol), N_A_ represents Avogadro constant, E represents optical power (W), λ represents incident optical wavelength (nm), h represents Planck constant (J⋅s) and c represents the speed of light (m/s).

**5. Photocatalytic H_2_O_2_ generation experiment**

The photocatalytic production of H_2_O_2_ was carried out under a 300W Xe lamp. Specifically, 10mg of catalyst was dispersed in a quartz reactor containing 45ml of water, followed by the addition of 5% ethanol as a sacrificial agent. Stir for half an hour in an air environment and darkness to achieve adsorption equilibrium. Then, turn on the Xe lamp and take samples every 15 min for one hour. The filtrate is filtered through a 0.22um filtration membrane. The detection of H_2_O_2_ was carried out using the iodometry method. 1ml of the solution was mixed with 0.5ml of 0.4M KI solution and 0.5ml of 0.1M C_8_H_5_KO_4_ solution and the color was developed for half an hour. Measure the absorbance at 350nm using a UV-vis spectrophotometer and compare it with the standard curve to obtain the H_2_O_2_ concentration.

**6. Density functional theory (DFT) calculations**

For the execution of all density functional theory (DFT) calculations, the Vienna Ab Initio Simulation Package (VASP) was utilized, employing the generalized gradient approximation (GGA) with the Perdew-Burke-Ernzerhof (PBE) functional formulation. Utilizing the projected augmented wave (PAW) potential framework, the ionic cores were modeled while valence electrons were considered. The electronic states were represented through a plane wave basis set, with a kinetic energy cutoff set at 450 eV. Partial occupancies of the Kohn-Sham or bitals were permitted through the application of the Gaussian smearing method, utilizing a smearing width of 0.05 eV. The 3 × 3 × 1 Monkhorst Pack grid was used to sample the Brillouin region. After static self-consistency was completed, the electrostatic potential and differential charge density were calculated.

**7. The IEF strength calculation method**

The IEF strength equation is as follows:

$$E={(\frac{-2V_{s}\rho}{\varepsilon\varepsilon_{0}})}^{1/2}$$

where E is the value of IEF. 𝑉𝑠and ρ are the surface voltage and surface charge density; 𝜀 and 𝜀_0_ are the low-frequency and vacuum dielectric constants, respectively. Therefore, 𝜀 and 𝜀_0_ are two constants. So the IEF is mainly determined by the surface voltage and the charge density. The surface voltage can be measured by open circuit voltage. The ρ can be tested and calculated by the Zeta potential according to the following equation :

$$\rho=\sqrt{8kT\varepsilon\varepsilon_{0}n}\sin h(\frac{ze_{0}V_{s}}{2kT})$$

For the 𝑉𝑠, there is an approximately equation :

$$V_{s}=\zeta\left( 1+\frac{D}{a_{1}} \right)e^{kD}$$

$\zeta$ is Zeta potential, $k$ is the Boltzmann constant, $T$ is absolute temperature, $n$ is Number of electrolytes per unit volume, $e_{0}$ is electron charge, $z$ is electrolyte valence, $a_{1}$ is Particle Stokes radius, is Debye length, $D$ is distance from Sliding Layer to Particle Surface. From the above equations, the built-in electric field (E) can be approximately concluded into a function of surface potential 𝑉𝑠 and Zeta potential $\zeta$:

$$E={(AV_{s}\sin h(\frac{ze_{0}\zeta(1+\frac{D}{a_{1}})e^{kD}}{2kT}))}^{1/2}$$

In summary, the surface voltage as well as the charge density are the main factors affecting the magnitude of the IEF strength. IEF will increase with the increase of 𝑉𝑠 and $\zeta$.


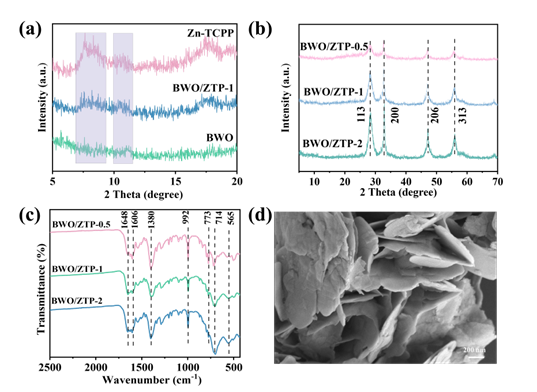


**Fig. S1.** (a) XRD local enlarged of BWO, Zn-TCPP and BWO/ZTP-1; (b) XRD patterns of BWO/ZTP-0.5, BWO/ZTP-1 and BWO/ZTP-2; (c) FT-IR spectra of BWO/ZTP-0.5, BWO/ZTP-1 and BWO/ZTP-2; (d) SEM of BWO/ZTP-1.


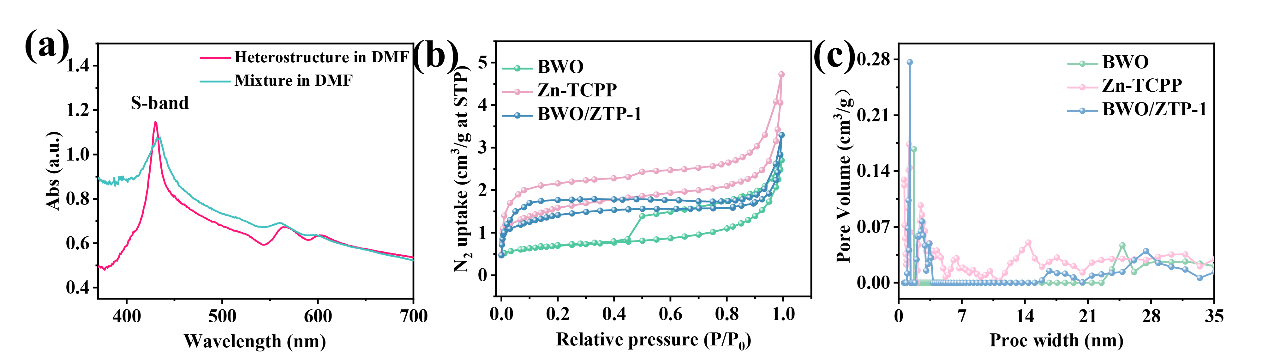


**Fig. S2.** (a) UV−vis absorption for physical mixture and heterojunction of Zn-TCPP and BWO dispersed in DMF; (b) N_2_ adsorption–desorption isotherms and (c) pore-size distributions of the BWO, Zn-TCPP and BWO/ZTP-1.


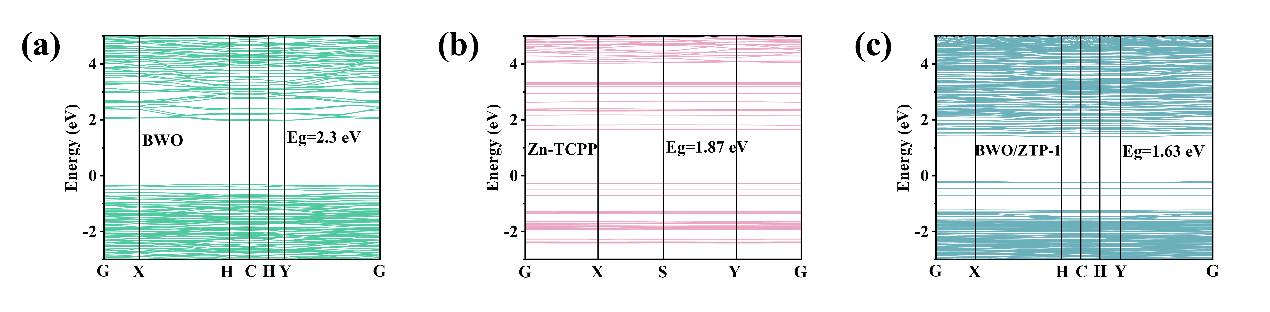


**Fig. S3.** (a-c) Band structure diagrams of BWO, Zn-TCPP and BWO/ZTP-1.


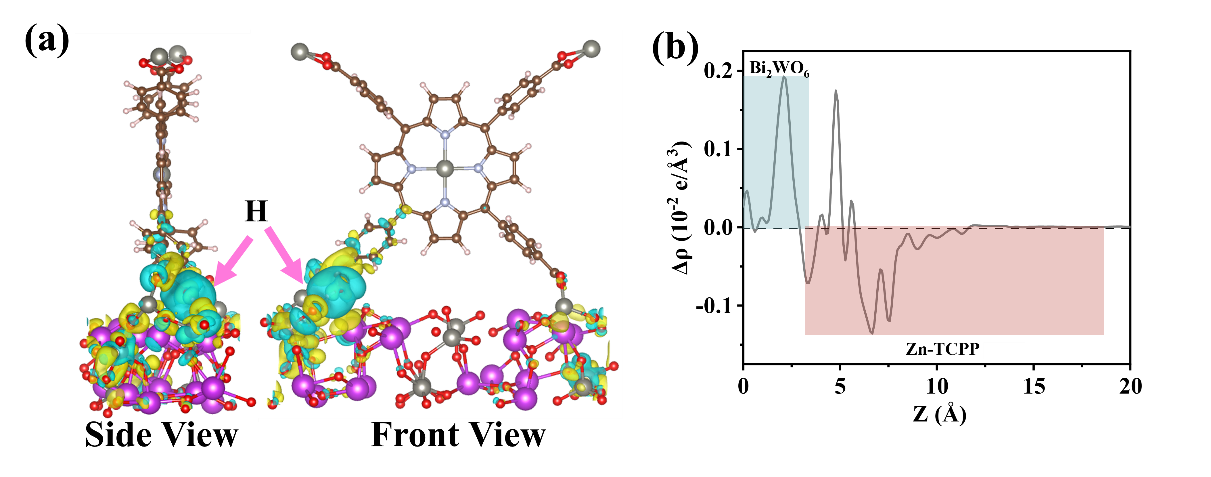


**Fig. S4.** (a) Differential charge density plot of H* adsorption sites of BWO/ZTP-1; (b) planar-averaged electron density difference Δρ (Z) for BWO/ZTP-1.


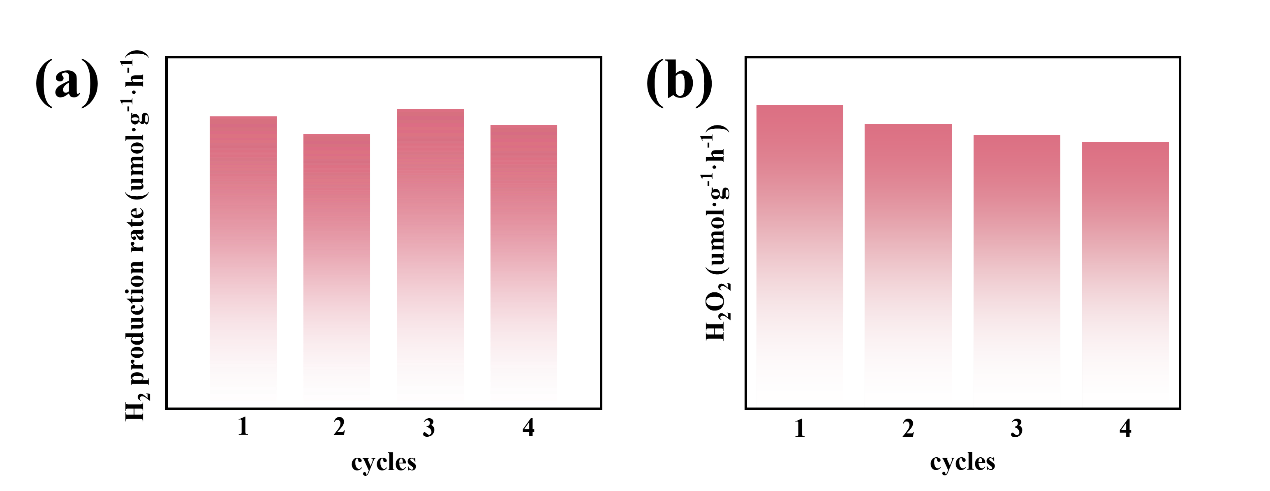


**Fig. S5.** (a) Cycle H_2_ and (b) H_2_O_2_ production experiment of BWO/ZTP-1.


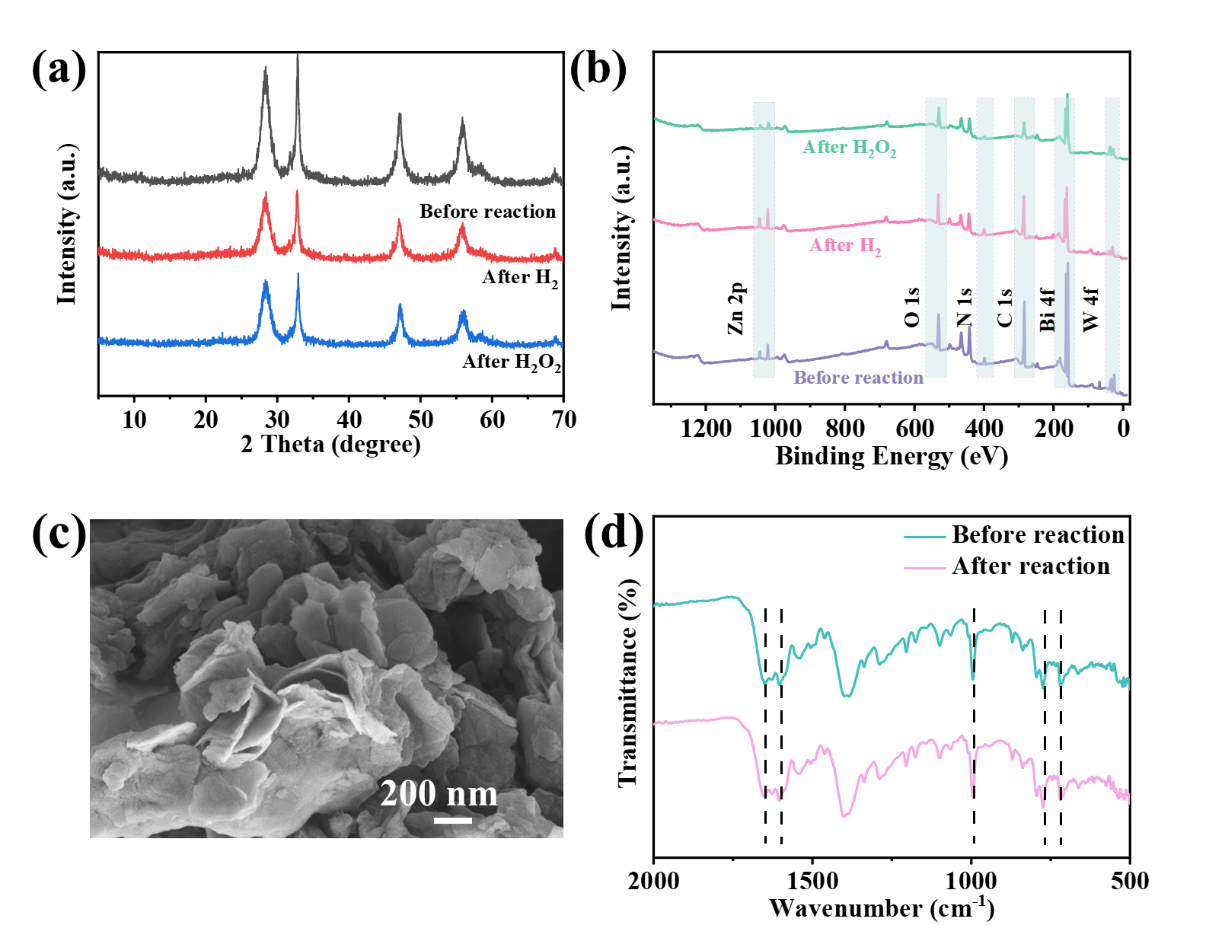


**Fig. S6.** (a) XRD and (b) XPS spectra; (c)SEM and (d) FT-IR before and after BWO/ZTP-1 reaction.


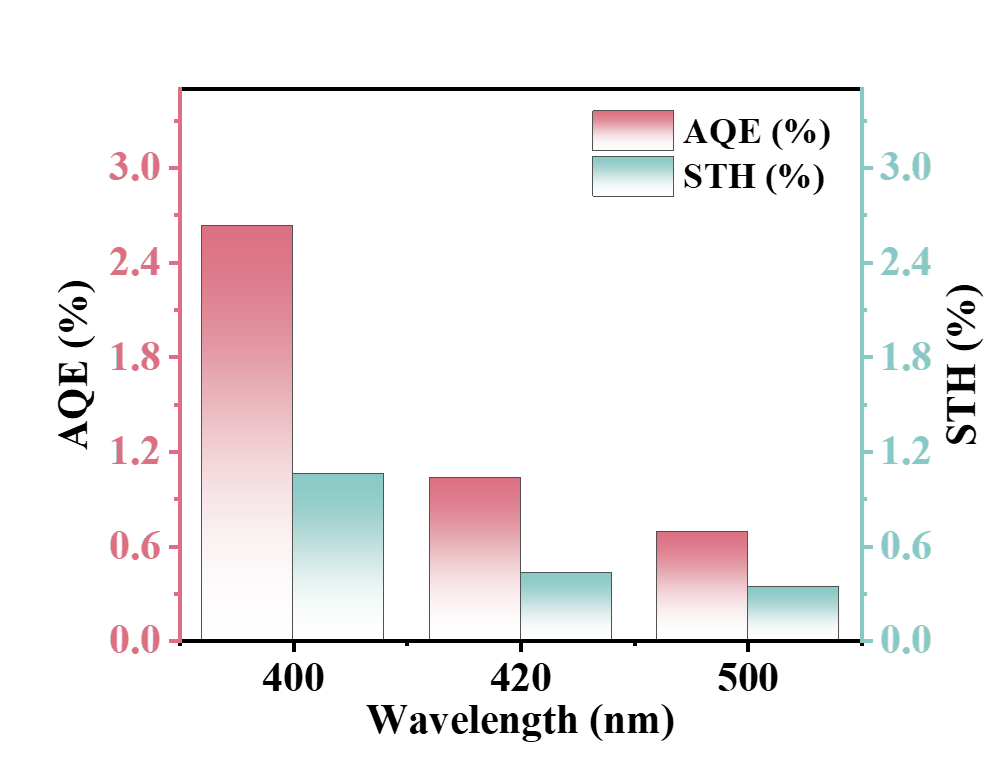


**Fig. S7.** AQE and STH for BWO/ZTP-1.

**Fig. S8.** Photocatalytic production of H_2_O_2_ by BWO/ZTP-1 under different conditions.

**Fig. S9.** The hydrogen production rate of BWO/ZTP-1 when different amounts of sacrificial agents are added.

**Table S1** Structure parameters of BWO, Zn-TCPP and BWO/ZTP-1.

| **Material** | **BET surface area (m^2^_·_g^-1^)** | **Total pore volume (cm^3^_·_g^-1^)** |
| --- | --- | --- |
| BWO | 17.35 | 0.094 |
| Zn-TCPP | 125.94 | 0.138 |
| BWO/ZTP-1 | 112.02 | 0.084 |

**Table S2** The IEF intensity of different materials

| **Material** | **IEF improvement multiple** | **Ref.** |
| --- | --- | --- |
| BWO/ZTP-1 | 2.1-2.5 | This work |
| BBW | 1.57-2.02 | [1] |
| ZnTCPP/C_60_-EDA | 1.86-4.08 | [2] |
| 0.5g‐SrTiO_3_‐PCN | 1.58-1.6 | [3] |
| ZnIn2S_3.5_Se_0.5_ | 1-1.83 | [4] |
| Na-PCN | 1.92 | [5] |
| PTrSO-2 | 1.04 | [6] |

**Table S3.** The average lifetimes of the BWO, Zn-TCPP and BWO/ZTP-1.

| **Samples** | **τ_1_ (ns)** | **τ_1_ (ns)** | **τ_ave_ (ns)** |
| --- | --- | --- | --- |
| BWO | 0.45 (57.21%) | 1.55 (42.79%) | 0.40 |
| Zn-TCPP | 0.68 (82.93%) | 2.25 (17.07%) | 0.49 |
| BWO/ZTP-1 | 1.05 (32.72%) | 3.66 (67.28%) | 1.05 |

**Table S4** Comparison of photocatalytic hydrogen evolution data of BWO-based photocatalysts reported in recent years.

| **Photocatalysts** | **Mass of photocatalyst** | **Light source** | **Sacrificial agents** | **Cocatalyst** | **Mass of cocatalyst** | **H_2_ Production rate** | **Ref.** |
| --- | --- | --- | --- | --- | --- | --- | --- |
| BWO/Zn-TCPP | 10 mg | 300 W Xe lamp, λ≥ 420 nm | AA | Pt | 1.0 wt% | 2343.3 μmol·g^-1^·h^-1^ | This work |
| BS15BWO | 100 mg | 250 W Xe lamp  λ≥ 420 nm | TEOA | — | — | 920 μmol·g^-1^·h^-1^ | [7] |
| Pt/S1BWO | 50 mg | 150 W Xe lamp, λ≥ 420 nm | TEOA | — | — | 250 μmol·g^-1^·h^-1^ | [8] |
| I-BWO | 20 mg | 350 W Xe lamp, λ≥ 420 nm | TEOA | — | — | 82.53 μmol·g^-1^·h^-1^ | [9] |
| BWO NPs | 20 mg | 300 W Xe lamp, λ≥ 420 nm | TEOA | Pt | 1.0 wt% | 148.4 μmol·g^-1^·h^-1^ | [10] |
| g-C_3_N_4_/Bi@Bi_2_WO_6_ | 50 mg | 300 W Xe lamp, | TEOA | Pt | 3.0 wt% | 1219.1 μmol·g^-1^·h^-1^ | [11] |
| Gr–BWO-T | 20 mg | 300 W Xe lamp,  λ≥ 420 nm | Methanol | — | — | 159.2 μmol·g^-1^·h^-1^ | [12] |
| Bi_2_WO_6_/ZnIn_2_S_4_ | 20 mg | 150 W Xe lamp  λ≥ 420 nm | Methanol | — | — | 131.8 μmol·g^-1^·h^-1^ | [13] |
| 2D Bi_2_WO_6_ nanosheets | 10 mg | 300 W Xe lamp | EDTA | Pt | 1.0 wt% | 56.9 μmol·g^-1^·h^-1^ | [14] |
| SUC-Bi_2_WO_6_ | 50 mg | 300 W Xe lamp, λ≥ 420 nm | Methanol | Pt | 3.0 wt% | 5.6 μmol·g^-1^·h^-1^ | [15] |

**Table S5 AQE data of BWO-based and Zn-TCPP-based photocatalysts reported in recent years.**

| **Material** | **AQE (420nm)** | **Ref.** |
| --- | --- | --- |
| BWO/ZTP | 1.03% | This work |
| CeO_2_/ZnTCPP | 0.57% | [16] |
| ZnTCPP-1 | 0.18% | [17] |
| ZnTCPP/C_60_-EDA | 0.78% | [2] |
| ZnPPO-50 | 0.19% | [18] |
| BCN-N-SA-10 | 0.376% | [19] |
| Bi_2_WO_6_/ZnIn_2_S_4_ | 0.53% | [20] |
| BWO-7 | 3.1% | [21] |
| BiWOH-70 | 1.09% | [22] |

**Reference**

[1] X. Deng, P. Chen, R. Cui, W. Huang, Y. Wu, X. Wang, C. Deng, Tip effect and structural disordering in Bi_2_WO_6_ for enhanced piezo-photocatalytic nitrogen oxidation to nitric acid, App. Catal. B: Environ. 339 (2023) 123148.

[2] J. Jing, J. Li, Y. Su, Y. Zhu, Non-covalently linked donor-acceptor interaction enhancing photocatalytic hydrogen evolution from porphyrin assembly, Appl. Catal. B Environ. 324 (2023) 122284.

[3] Z. Wei, J. Yan, W. Guo, W. Shangguan, Nanoscale lamination effect by nitrogen-deficient polymeric carbon nitride growth on polyhedral SrTiO_3_ for photocatalytic overall water splitting: Synergy mechanism of internal electrical field modulation, Chin. J. Catal. 48 (2023) 279-289.

[4] X. Yi, S. Zhang, H. Shen, B. Li, L. Yang, W. Dai, R. Song, J. Zou, S. Luo, Atomic sulfur dissimilation remolding ZnIn_2_S_4_ nanosheets surface to enhance built-internal electric field for photocatalytic CO_2_ conversion to syngas, App. Catal. B: Environ. 338 (2023) 123003.

[5] L. Jian, Y. Dong, H. Zhao, C. Pan, G. Wang, Y. Zhu, Highly crystalline carbon nitrogen polymer with a strong built-in electric fields for ultra-high photocatalytic H_2_O_2_ production, App. Catal. B: Environ. 342 (2024) 123340.

[6] F. Yu, Z. Zhu, S. Wang, J. Wang, Z. Xu, F. Song, Z. Dong, Z. Zhang, Novel donor-acceptor-acceptor ternary conjugated microporous polymers with boosting forward charge separation and suppressing backward charge recombination for photocatalytic reduction of uranium (VI), App. Catal. B: Environ. 301 (2022) 120819.

[7] R. Bariki, Y.P. Bhoi, S.K. Pradhan, S. Panda, S.K. Nayak, K. Das, D. Majhi, B.G. Mishra, Oxygen defect rich Bi_2_S_3_/SnS_2_/Bi-self doped Bi_2_W_2_O_9_ multijunction photocatalyst for enhanced degradation of methyl parathion and H_2_ evolution, Sep. Purif. Technol. 324 (2023) 124509.

[8] Z. Li, K. Wang, J. Zhang, Y. Chang, E. Kowalska, Z. Wei, Enhanced Photocatalytic Activity of Hierarchical Bi_2_WO_6_ Microballs by Modification with Noble Metals, Catalysts 12(2) (2022).

[9] W. Liu, K. Qi, Y. Wang, F. Wen, J. Wang, Halogen-induced polymorphic Bi_2_WO_6_-xhalogen2x with highly photocatalytic performance and mechanism investigation, Appl. Surf. Sci. 600 (2022) 154160.

[10] L. Wang, C. Guo, F. Chen, J. Ning, Y. Zhong, Y. Hu, pH-induced hydrothermal synthesis of Bi_2_WO_6_ nanoplates with controlled crystal facets for switching bifunctional photocatalytic water oxidation/reduction activity, J. Colloid Interface Sci. 602 (2021) 868-879.

[11] W. Quan, J. Bao, X. Meng, Y. Ning, Y. Cui, X. Hu, S. Yu, H. Tian, 2D/2D Z-scheme photocatalyst of g-C_3_N_4_ and plasmonic Bi metal deposited Bi_2_WO_6_: Enhanced separation and migration of photoinduced charges, J. Alloys Compd. 946 (2023) 169396.

[12] Z. Sun, J. Guo, S. Zhu, L. Mao, J. Ma, D. Zhang, A high-performance Bi_2_WO_6_–graphene photocatalyst for visible light-induced H_2_ and O_2_ generation, Nanoscale 6(4) (2014) 2186-2193.

[13] A. Chachvalvutikul, T. Luangwanta, S. Pattisson, G.J. Hutchings, S. Kaowphong, Enhanced photocatalytic degradation of organic pollutants and hydrogen production by a visible light–responsiveBi_2_WO_6_/ZnIn_2_S_4_ heterojunction, Appl. Surf. Sci. 544 (2021) 148885.

[14] S. Wu, J. Sun, Q. Li, Z.D. Hood, S. Yang, T. Su, R. Peng, Z. Wu, W. Sun, P.R.C. Kent, B. Jiang, M.F. Chisholm, Effects of Surface Terminations of 2D Bi_2_WO_6_ on Photocatalytic Hydrogen Evolution from Water Splitting, ACS Appl. Mater. Interfaces 12(17) (2020) 20067-20074.

[15] H. Huang, R. Cao, S. Yu, K. Xu, W. Hao, Y. Wang, F. Dong, T. Zhang, Y. Zhang, Single-unit-cell layer established Bi_2_WO_6_ 3D hierarchical architectures: Efficient adsorption, photocatalysis and dye-sensitized photoelectrochemical performance, Applied Catalysis B: Environmental 219 (2017) 526-537.

[16] Y. Wang, B. Xu, H. Yang, S. Fan, M. Zhang, T. Ohno, Construction of CeO_2_/ZnTCPP heterojunction with enhanced photocatalytic hydrogen production performance, Mater. Lett. 360 (2024) 135925.

[17] Z. Jin, J. Zhang, J. Qiu, Y. Hu, T. Di, T. Wang, Nitrogen vacancy-induced spin polarization of ultrathin zinc porphyrin nanosheets for efficient photocatalytic CO_2_ reduction, J. Colloid Interface Sci. 652 (2023) 122-131.

[18] X. Xu, X. Feng, W. Wang, K. Song, D. Ma, Y. Zhou, J.-W. Shi, Construction of II-type and Z-scheme binding structure in P-doped graphitic carbon nitride loaded with ZnO and ZnTCPP boosting photocatalytic hydrogen evolution, J. Colloid Interface Sci. 651 (2023) 669-677.

[19] J. Li, X. Ju, X. Feng, Y. Zhang, G. Huang, D. Ma, X. Zhao, X. Xu, J.W. Shi, Constructing Z-scheme between graphite nitride carbon and supramolecular zinc porphyrin to promote photocatalytic H_2_ evolution, J. Colloid Interface Sci. 690 (2025) 137284.

[20] D. Sarkar, J. Pramanik, S. Ghosal, S. Paul, P.K. Giri, A. Datta, S.S. Sinha, G. Roymahapatra, S. Jain, R.K. Singh, S. Ghosh, Synergistic Effect of Atomic‐Scale Interface Engineering and Built‐In Electric Field at S‐Scheme Bi_2_WO_6_/ZnIn_2_S_4_ Heterojunctions for Photocatalytic Hydrogen Evolution, Small 21(33) (2025).

[21] L. Wang, C. Guo, F. Chen, J. Ning, Y. Zhong, Y. Hu, pH-induced hydrothermal synthesis of Bi_2_WO_6_ nanoplates with controlled crystal facets for switching bifunctional photocatalytic water oxidation/reduction activity, J. Colloid Interface Sci. 602 (2021) 868-879.

[22] S. Liu, N. Song, Y. Zhu, F. He, M. Zhang, K. Zhang, Rational design of strongly coupled inorganic/organic bismuth tungstate/hydrogen-bonded organic frameworks heterojunctions with optimized interface interactions for enhanced photocatalytic performance, J. Colloid Interface Sci. 700 (2025) 138362.
