# Supplementary figures and images for: Fabricating Inorganic/Organic S-Scheme Heterojunction for Efficient Photocatalytic Production of H2 and H2O2
Source: Research (Wash D C). 2026 Mar 3;9:1166. doi: 10.34133/research.1166 (PMC12954277; doi:10.34133/research.1166)

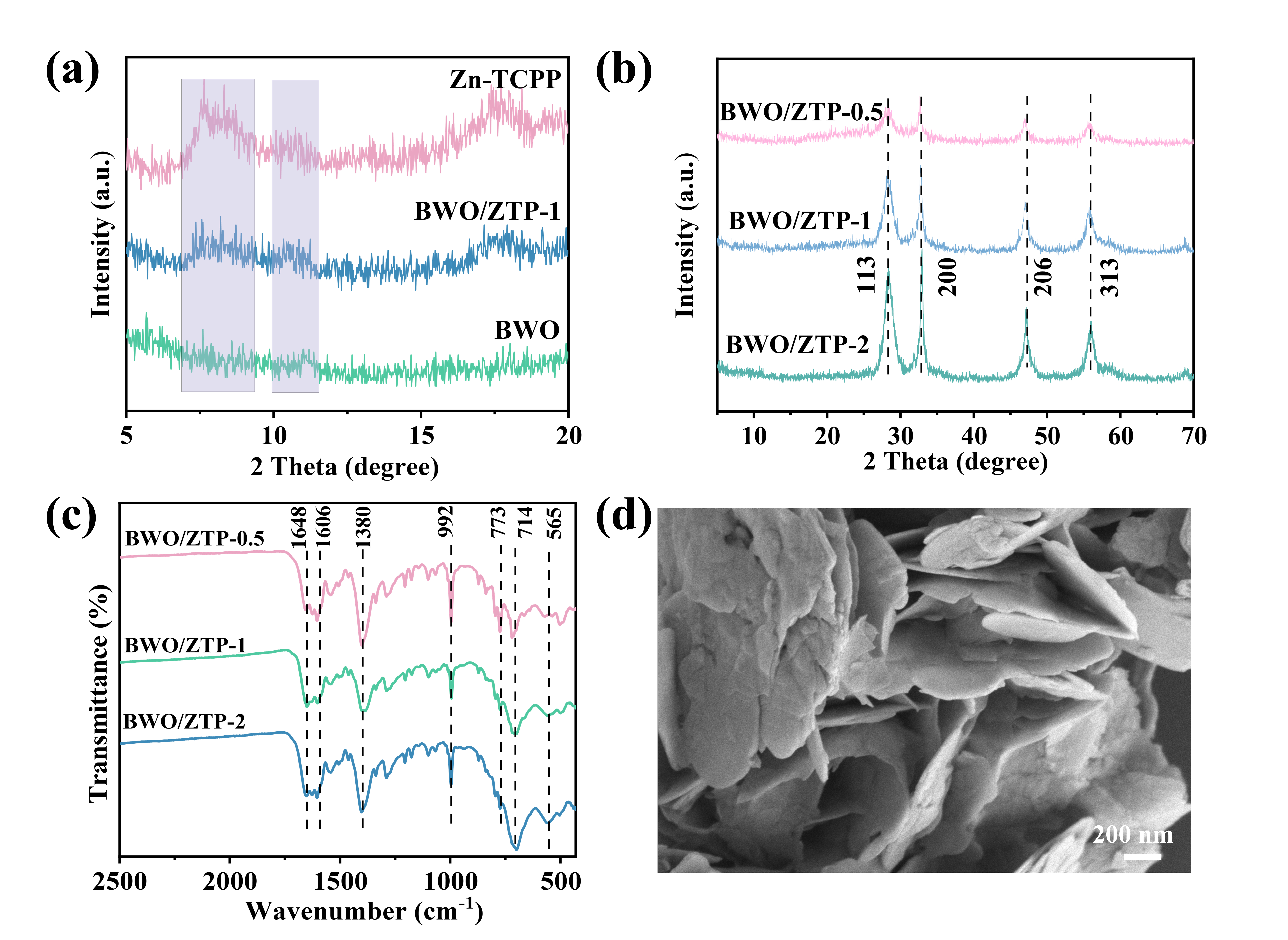

Supplement: Supplementary 1 — Supplementary Text Figs. S1 to S9 Tables S1 to S5 [file research.1166.f1.zip › Fig. S1.tif]

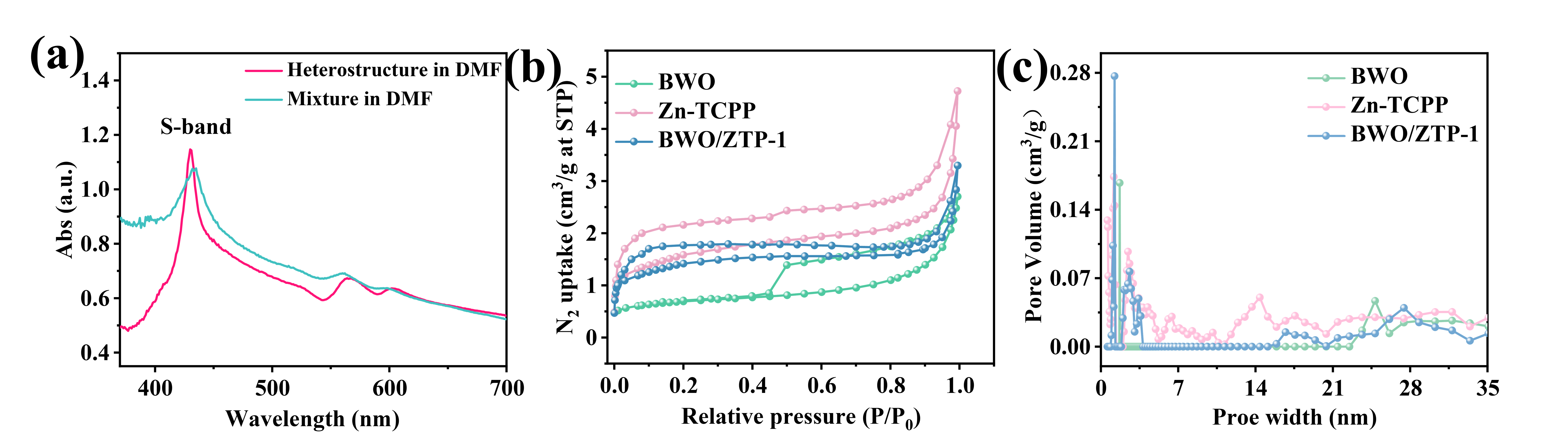

Supplement: Supplementary 1 — Supplementary Text Figs. S1 to S9 Tables S1 to S5 [file research.1166.f1.zip › Fig. S2.tif]

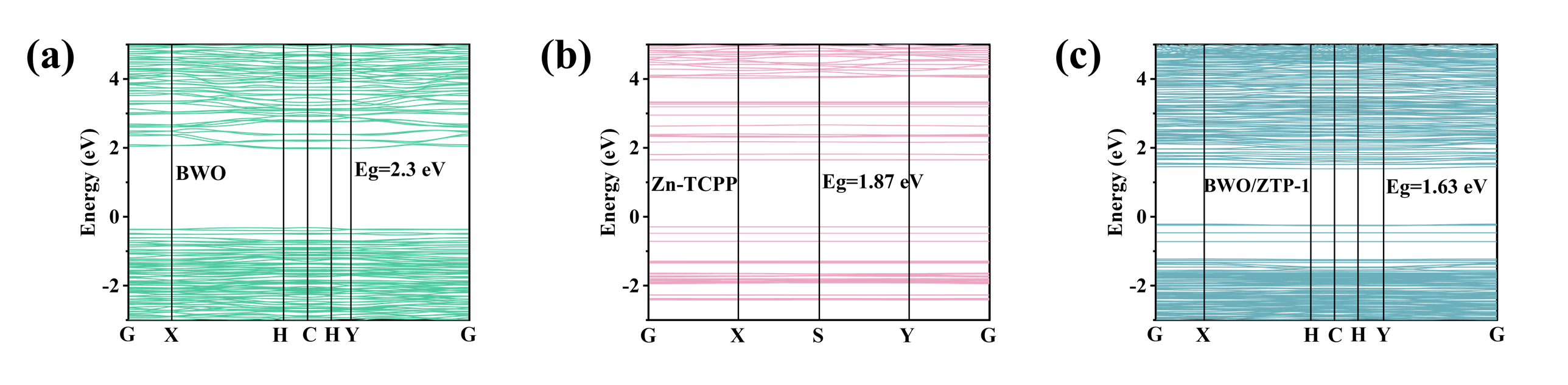

Supplement: Supplementary 1 — Supplementary Text Figs. S1 to S9 Tables S1 to S5 [file research.1166.f1.zip › Fig. S3.tif]

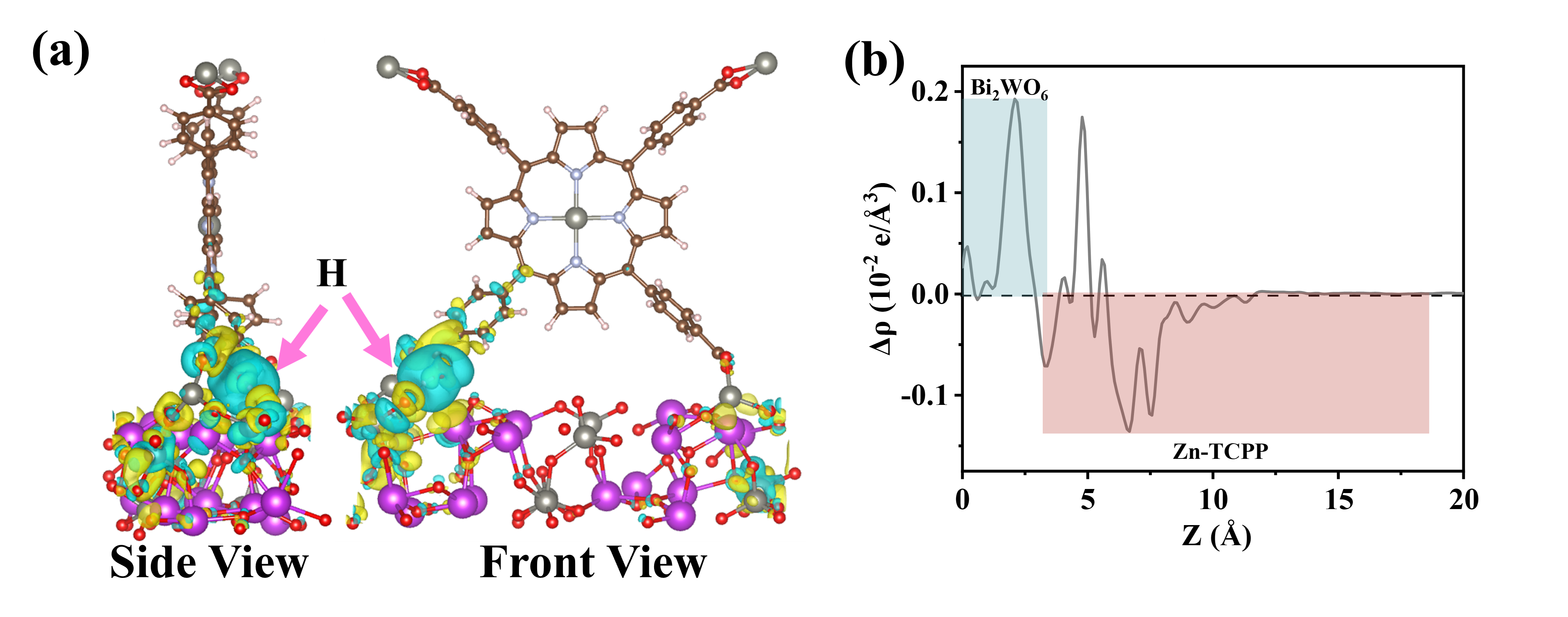

Supplement: Supplementary 1 — Supplementary Text Figs. S1 to S9 Tables S1 to S5 [file research.1166.f1.zip › Fig. S4.tif]

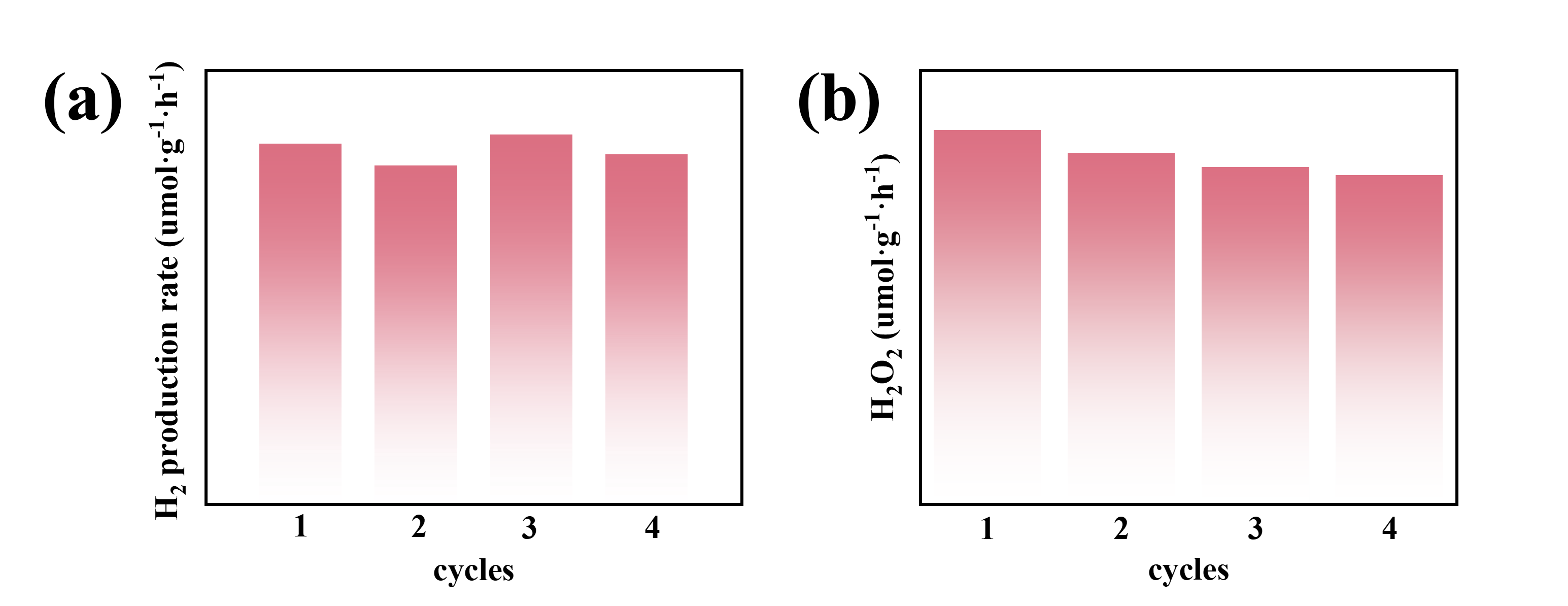

Supplement: Supplementary 1 — Supplementary Text Figs. S1 to S9 Tables S1 to S5 [file research.1166.f1.zip › Fig. S5.tif]

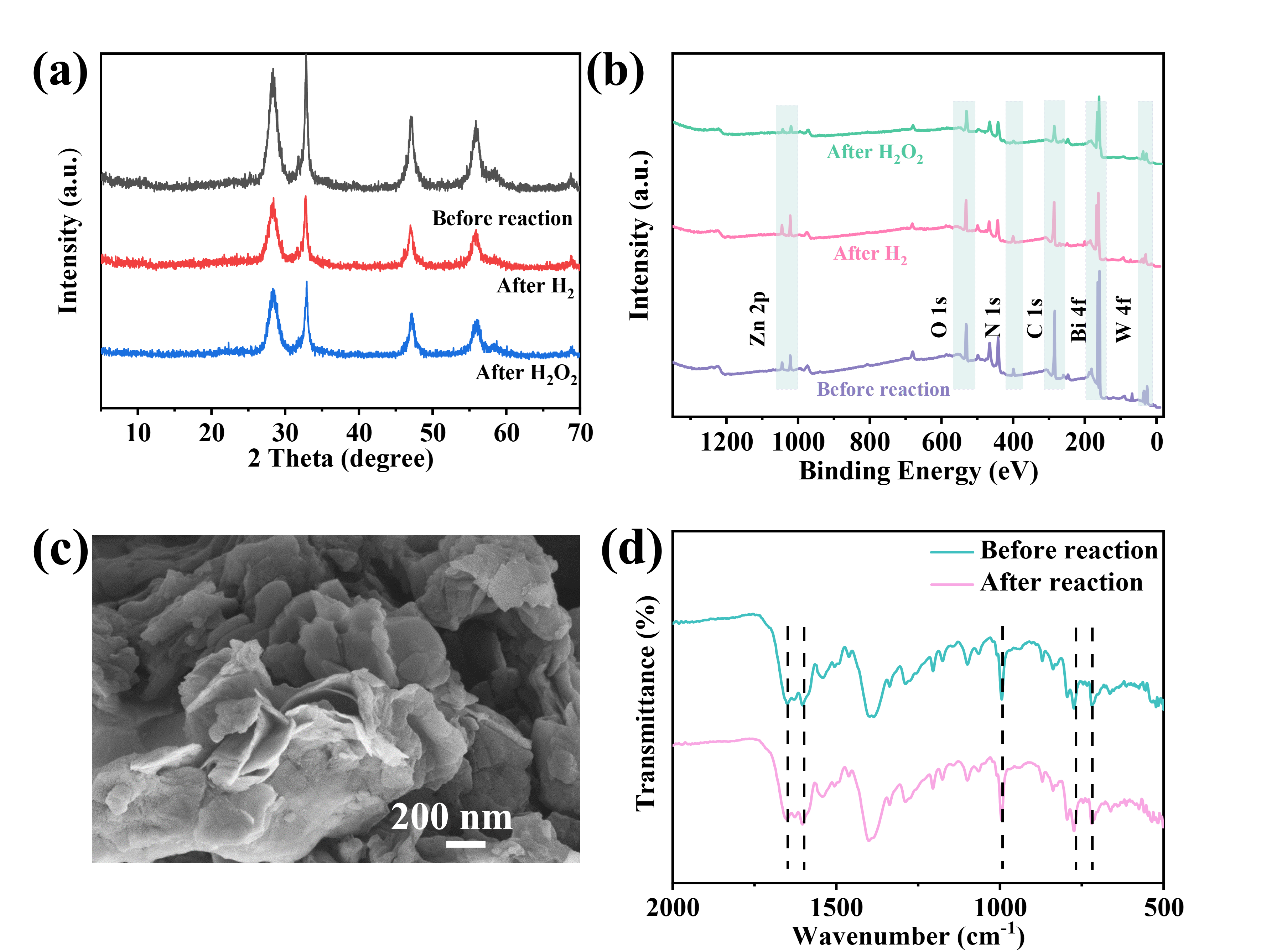

Supplement: Supplementary 1 — Supplementary Text Figs. S1 to S9 Tables S1 to S5 [file research.1166.f1.zip › Fig. S6.tif]

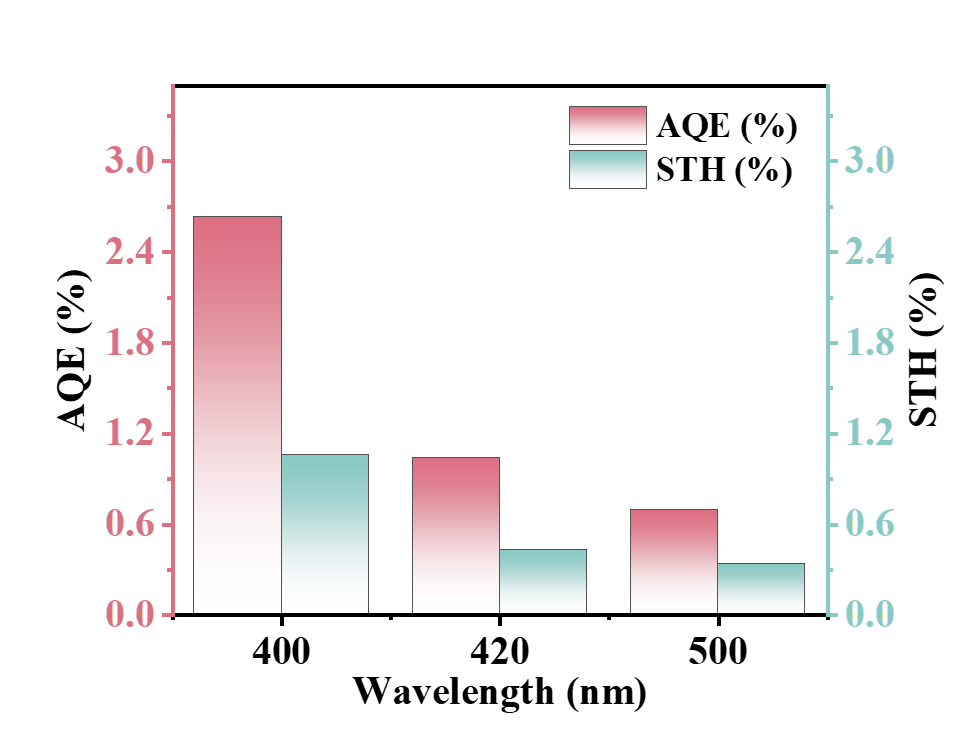

Supplement: Supplementary 1 — Supplementary Text Figs. S1 to S9 Tables S1 to S5 [file research.1166.f1.zip › Fig. S7.tif]

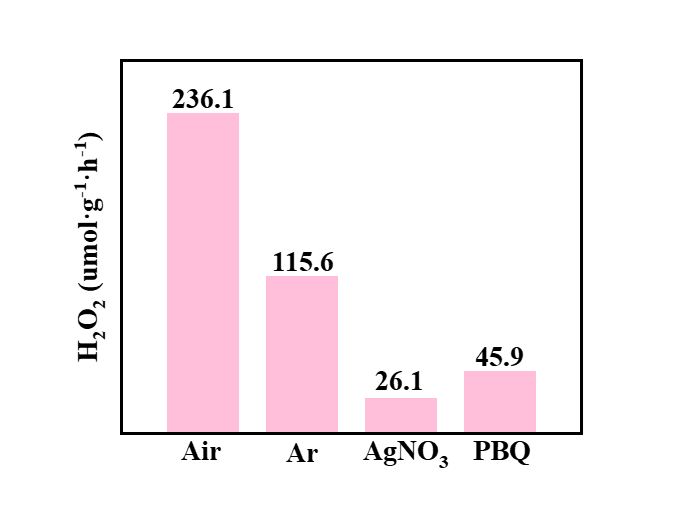

Supplement: Supplementary 1 — Supplementary Text Figs. S1 to S9 Tables S1 to S5 [file research.1166.f1.zip › Fig. S8.tif]

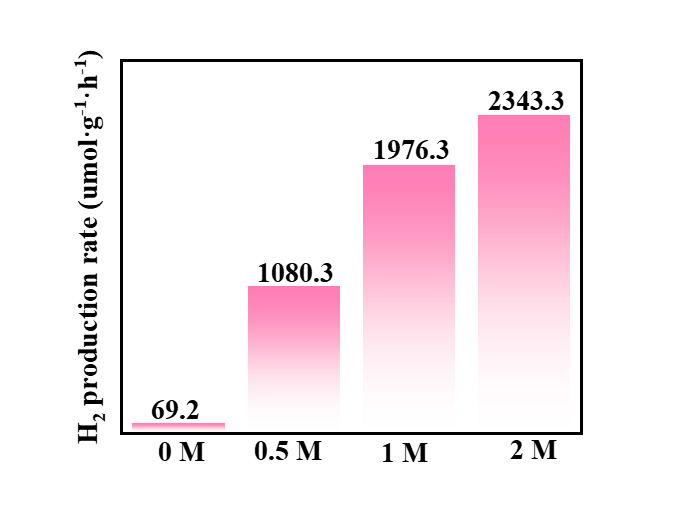

Supplement: Supplementary 1 — Supplementary Text Figs. S1 to S9 Tables S1 to S5 [file research.1166.f1.zip › Fig. S9.tif]
